# Sex-biased transcriptome in in vitro produced bovine early embryos

**DOI:** 10.1186/s13578-025-01459-x

**Published:** 2025-08-27

**Authors:** Meihong Shi, Guangsheng Li, Hannah Marie Araujo, Angie S. Lee, Jingzhi Zhang, Yoke Lee Lee, IISAGE Consortium, Nicole C. Riddle, Peggy R. Biga, Anne M. Bronikowski, Richard Meisel, James R Walters, Tony Gamble, Gerald S. Wilkinson, Erica Larschan, Ritambhara Singh, Ashley Webb, Soon Hon Cheong, Jingyue Ellie Duan

**Affiliations:** 1 Department of Animal Science, College of Agriculture and Life Science, Cornell University, Ithaca, NY USA; 2 Department of Clinical Science, College of Veterinary Medicine, Cornell University, Ithaca, NY USA

**Keywords:** Bovine blastocyst, Sex difference, Differential gene expression, Sex-biased alternative splicing, Isoform expression

## Abstract

**Background:**

Morphologic sex differences between males and females typically emerge after the primordial germ cell migration and gonad formation, although sex is determined at fertilization based on chromosome composition. A key debated sexual difference is the embryonic developmental rate, with in vitro produced male embryos often developing faster. However, the molecular mechanisms driving early embryonic sex differences remain unclear.

**Results:**

To investigate the transcriptional sex difference during early development, in vitro produced bovine blastocysts were collected and sexed by PCR. A significant male-biased development is consistently observed in expanded blastocysts. Ultra-low input RNA-seq analysis identified 837 DEGs, 1555 significantly sex-biased differential alternative splicing (AS), and 1151 differentially expressed isoforms (DEIs). Among all of the DEGs, there were 231 upregulated and 606 downregulated in males. Functional enrichment analysis revealed male-biased DEGs were associated with metabolic regulation, whereas female-biased DEGs were related to female gonad development, sex differentiation, inflammatory pathways, and TGF-beta signaling. Comparing X chromosome and autosome expression ratio, we found that female-biased DEGs contributed to the higher X-linked gene dosage, a phenomenon not observed in male embryos. Moreover, we identified the sex-biased transcription factors and RNA-bind proteins, including pluripotent factors such as *SOX21* and *PRDM14*, and splicing factors *FMR1* and *HNRNPH2*. Additionally, we revealed that the significantly sex-biased differential AS were predominantly skipped exons, and they could be mapped to 906 genes, with 59 overlapping with DEGs enriched in metabolic and autophagy pathways. By incorporating novel isoforms from long reads sequencing, the sex-biased DEIs were associated with 1017 genes. Functional analysis showed that female-biased DEIs were involved in the negative regulation of transcriptional activity, while male-biased DEIs were related to energy metabolism. Furthermore, we identified sex-biased differential exon usage in *DENND1B, DIS3L2, DOCK11, IL1RAPL2,* and *ZRSR2Y,* indicating their sex-specific regulation in early embryo development.

**Conclusion:**

This study provided a comprehensive analysis of transcriptome differences between male and female bovine blastocysts, integrating sex-biased gene expression, alternative splicing, and isoform dynamics. Our findings indicate that enriched metabolism processes in male embryos may contribute to the faster developmental pace, providing insights into sex-specific regulatory mechanisms during early embryogenesis.

**Supplementary Information:**

The online version contains supplementary material available at 10.1186/s13578-025-01459-x.

## Background

Assisted reproductive technologies (ARTs), particularly in vitro fertilization (IVF), have been widely used to improve livestock fertility, herd genetics, and production. One interesting observation in these technologies is the differing developmental pace between male and female embryos during the early stages of embryogenesis, even before gonad differentiation occurs [1, 2]. This difference has been observed in various species over decades, including humans [3–9], mice [10–12], cattle [1, 13, 14], pigs [15, 16], and sheep [17]. However, the findings are controversial, as some studies report no significant sex biases in blastocyst formation and birth count, particularly in human IVF embryos [18–20]. Culture conditions and experimental design, such as differences in IVF methods, culture medium supplements, and in vivo or in vitro developmental environment, may contribute to these discrepancies [21, 22].

Previous studies have identified some sex-specific differences in early embryos prior to gonadal differentiation, such as differential expression of imprinted and sex-linked genes, glucose metabolism related activities, mitochondrial DNA (mtDNA) copy numbers, and telomere lengths [23–28]. Recent advances in transcriptomics, including microarray and RNA sequencing (RNA-seq) using embryos fertilized by sex-sorted semen or in single embryos, have provided deeper insights into these sex-specific differences at the transcriptomic level [29–33]. For example, in vivo produced bovine male embryos show a different transcriptome profile [30, 31], having a higher expression of pluripotent factors and a lower expression of genes related to glucose transport and apoptosis compared to females [34]. Besides, single-cell RNA-seq in human embryos indicated a distinct transcriptomic dynamic between female and male embryos after embryonic genome activation [32]. Similarly, the pig embryos also showed distinct transcriptional differences between sexes with a dynamic compensation of X chromosome in the female embryos [33]. Moreover, a recent comparative transcriptome analysis between human and mouse embryos revealed conserved sex-biased expression across preimplantation stages, suggesting the establishment of the sex-specific protein–protein interaction networks involved in epigenetic reprogramming during embryogenesis [35]. Despite these findings, the underlying molecular mechanisms driving sex-specific differences in early embryogenesis remain poorly understood. Moreover, the use of embryos produced with sex-sorted semen introduces additional variability [34], as the presence of mixed sexed embryos can influence results. Thus, a comprehensive investigation of transcriptomic differences between in vitro produced male and female early embryos with precisely defined sex is essential to address the knowledge gap in sex-specific embryogenesis.

Regulation of transcription is mediated by multiple mechanisms, including transcription factors (TFs), TF-associated cofactors, and alternative splicing (AS). TFs are DNA-binding proteins that activate or suppress transcription, playing critical roles in gene regulation across evolution, development, and diseases [36]. Alternative splicing, controlled by spliceosome complex, allows a single gene to generate multiple functional isoforms [37], significantly expanding the transcriptome diversity. Although short-read RNA-seq technologies have been widely used to assemble the embryonic isoforms, their limited read length often leads to incomplete isoform annotation and ambiguous splice junction resolution [38]. In contrast, long-read sequencing with the Pacific Biosciences platform, representing the third generation of sequencing technology, can overcome the shortcomings of RNA-seq via the direct reading of full-length transcripts with low sequencing error rates [39]. In recent studies, long-read sequencing has enabled the identification of novel transcribed regions and novel splicing variants involved in preimplantation embryogenesis. For instance, a recent human study generated an isoform-resolved transcriptome of preimplantation development and identified novel isoforms transcribed from known or unannotated genes. Further integrative analysis revealed that human embryonic genome activation was associated with splicing disruption and transient upregulation of gene co-expression modules [40]. These findings highlight the importance of long-read sequencing in uncovering transcriptomic complexity during early embryonic development.

Beyond autosomal regulations, the regulation of genes located on sex chromosomes also has significant contributions to sex determination and sex-specific development. In mammals, the XY sex-determination system requires a balance of gene dosage between males (XY) and females (XX), and between sex chromosomes and autosomes [41]. Mechanisms such as X-chromosome inactivation (XCI) in females and X-chromosome upregulation (XCU) in both sexes are essential to maintain this balance and prevent gene dysregulation [42]. In early mammalian embryogenesis, the exact timing and the extent of these processes remain unclear. While Xist expression begins early in development, partial XCI has been observed [43], with some of the X-linked genes escaping the XCI silencing [44, 45]. Whether X dosage compensation contributes to the sex-specific embryo developmental differences in bovine embryos remains unclear.

In this study, we aim to uncover genomic differences between male and female bovine embryos produced in vitro. Using PCR-based single embryo sex determination and comprehensive RNA-seq analysis, we identified the differential gene expression (DEG) and associated regulators, examined the X-chromosome dosage compensation, characterized the sex-biased alternative splicing events, and analyzed isoform diversities between male and female blastocysts. Our findings reveal that male embryos developed faster, driven by upregulated metabolism activities compared to female embryos, while female embryos were more active in immune response, female-specific gonadal development, and protein processing.

## Materials and methods

### Embryo production and sample collection

Bovine embryos were produced from slaughterhouse ovaries using commercial media (IVF Bioscience, Falmouth, UK) according to the manufacturer protocols. Bovine ovaries were obtained from a slaughterhouse (Cargill, Wyalusing, PA) and transported to the lab in warm saline solution (0.9% Sodium chloride, Sigma-Aldrich) maintained between 23.5–30 °C within 2 h. Follicles (diameter: 2–8 mm) were aspirated using an 18-gauge hypodermic needle attached to an aspiration vacuum pump. Cumulus-oocyte complexes (COCs) with homogenous cytoplasm and surrounded by at least three layers of cumulus cells were selected and matured in BO-IVM™ medium (IVF Bioscience, Falmouth, UK) for 22 h at 38.5 °C in a humidified atmosphere with 5% CO_2_.

Matured COCs were transferred to BO-IVF™ medium (IVF Bioscience, Falmouth, UK) and cocultured with thawed semen at a final concentration of 1 × 10^6^ sperm/mL at 38.5 °C in a humidified atmosphere with 5% CO_2_. Fertilization was performed with cryopreserved semen from bulls of known fertility generously donated by Genex Cooperative. Semen preparation was conducted using BO-SemenPrep™ medium and recovered sperm concentration was determined with a sperm cell counter (Nucleocounter SP-100, ChemoMetec, Allerod, Denmark) then used for fertilization. To improve genetic diversity and biological robustness, semen from two bulls of the same genetic background was used in rotation each week. No difference in embryo developmental rates was observed between bulls. After 18 h of fertilization, presumptive zygotes were denuded by vortexing at 3200 rpm for 60 s twice in a wash medium (IVF Bioscience, Falmouth, UK). Zygotes with intact membranes were transferred into BO-IVC™ medium (IVF Bioscience, Falmouth, UK) and cultured in a humidified atmosphere (5% O_2_, 5% CO_2_, and 90%N_2_) at 38.5 °C for further blastocyst collection.

**Fig. 1:**
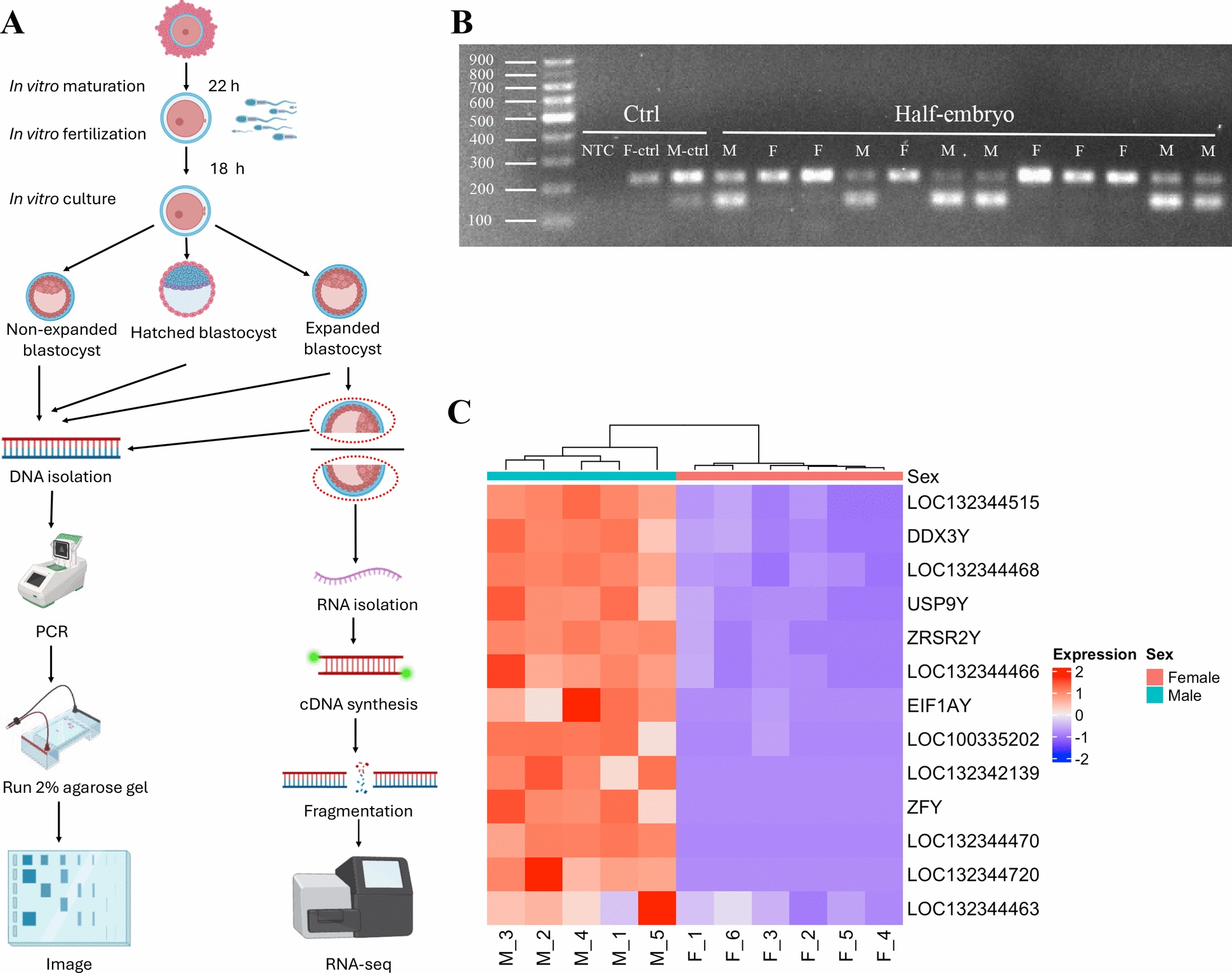
Overview of embryo sex determination. **A** Schematic overview of the experimental setup. Expanded blastocysts were cut into two halves evenly, with each half containing inner cell mass (ICM), trophectoderm (TE), and zona pellucida. One half was used for RNA-seq, and the other half was used for PCR-based sex determination. **B** Agarose gel of amplified gDNA of half blastocysts. Each lane is one control sample or a half-cut embryo; NTC: no-template control; F-ctrl: gDNA from bovine mammary alveolar cells (MAC-T cells); M-ctrl: gDNA from bovine testis tissues. Identification of the autosomal amplicon (217 bp) located above and the Y-specific amplicon (143 bp) located below. **C** Heatmap of all male-specific DEGs located on Y-chromosome in all RNA-seq samples. Each column represented an individual RNA-seq sample, and the hierarchical clustering along the X axis represented sample similarity based on the selected Y-linked gene expression. The color scale in the heatmap represents the z-score of normalized gene expression

To minimize variability associated with developmental stage [46], only expanded blastocysts on Day 7 and Day 8 were collected to be cut into two halves for sex determination and RNA-seq. After determining embryo sex, two half-cut expanded blastocysts of the same sex were pooled as one replicate. In total, 5 male and 6 female replicates were obtained. This pooling strategy was necessary due to the limited embryo number and aimed to increase RNA yield while still capturing representative transcriptomic signatures. Moreover, to increase the statistical power of sex ratio analysis, a total of 444 blastocysts including expanded and hatched blastocysts on Day 7, and non-expanded, expanded, and hatched blastocysts on Day 8 were collected for PCR-based sex determination. Embryos were washed three times with DPBS (Life Technology, USA), collected individually in PCR tubes, and snap-frozen at − 80 °C for later sex determination. Expanded blastocysts were placed individually in 10ul drops of DPBS covered with mineral oil in 35 mm culture dishes after a DPBS wash and evenly divided into two halves with a microblade under the microscope. Each half contained both the inner cell mass and trophectoderm (Fig. 1A). Cut embryos were stored separately at − 80 °C for future use.

### Sex determination

Embryo sexing followed a modified protocol [47]. Briefly, dissected embryos were lysed with 5 µL of cell lysis buffer with Proteinase K (1: 50 dilution, SingleShot Cell Lysis kit, 1725081, Bio-rad) and 100 µg/mL RNase (T3018L, New English Biolab), incubated at room temperature (RT) for 10 to 20 min, followed by 5 min at 37 °C and 5 min at 75 °C.

For PCR, 12.5 µL of One*Taq*^®^ Quick-Load^®^ 2 × Master Mix (M0486L, New England Biolabs) was mixed with 0.4 µl of 10 μM primers and 7.1 µl of nuclease-free water. The first round of PCR amplified a Y-linked DNA region using following conditions: 95 °C for 5 min; 20 cycles of 95 °C for 15 s, 58 °C for 15 s, and 72 °C for 15 s; then 10 min at 72 °C. For the second round, 1 μL of 4 μM autosomal primers were added, and an additional 17 PCR cycles were performed under the same conditions. Primer sequences were listed in Table S1.

PCR products (5 μL) were mixed with 1 μL of 6 × DNA loading dye (R0611, Thermo Scienticic) and loaded on 2% (wt/vol) agarose gels stained with SYBR Safe DNA gel Stain (S33102, Thermo Scienticic) for electrophoresis. Embryos were classified as males if the Y-linked and autosomal amplicons were both present and as females if only the autosomal amplicon was detected. A Pearson Chi-square test was performed using R for statistical comparisons for the numbers between female and male embryos.

### RNA-seq library construction

Given the limited sexed embryo sample obtained from each IVF week, two half-cut embryos with the same sex were pooled as a replicate. This strategy aimed to reduce individual embryo bias and ensure that each sample more accurately reflected group-level sex-specific transcriptomic differences. Five male and six female samples were used for RNA-seq library construction. Libraries were constructed using SMART-Seq^®^ HT PLUS Kit (R400748, Taraka). Briefly, pooled embryos were lysed in 1 × Lysis buffer with RNase inhibitor, and full-length double-strand cDNA was synthesized and amplified with 15 cycles with a one-step master buffer. The cDNA was quantified by Fragment Analyzer 5200 (Agilent) and purified with AMPure XP beads (Beckman Coulter). For each replicate, 8 ng of cDNA was fragmented, stem-loop adapter was added, and followed by 13 cycles of PCR for library amplification with unique dual indexes. Libraries were quality controlled (QC) and sequenced at Novogene Inc. (Sacramento, CA, USA), using NovoSeq PE150 platform.

### RNA-seq analysis

The adaptor removal and QC of the raw sequencing reads were performed using fastp (v 0.20.0) [48]. Reads with a percentage of low-quality base (quality score < 20) > 40% were removed. Reads with length < 30 bp or with high N content (> 5%) were also removed in this study. The cow reference genome (ARS-UCD 2.0) was downloaded from NCBI database, and clean reads were aligned to the reference genome using STAR (v 2.7.10b) [49] allowing no more than 3 mismatches. The raw read counts for each gene were extracted using featureCounts (v 2.0.3) [50], and the gene expression level was normalized by transcripts per million (TPM) using TPMCalculator [51]. To check the sample correlations, the principal component analysis (PCA) was conducted using the top 500 variable genes.

For identifying the differentially expressed genes (DEGs) between sexes, the raw read count matrix was imported in DEseq2 [52] R package. The pair-wise comparison was performed between female and male embryos, and DEGs were determined using the following criteria: |Fold Change (FC) > 1.5| and FDR < 0.1. To correct potential batches, including those related to collection weeks and bull semen, samples collection week was included as a covariate in the DESeq2 differential expression analysis. Additionally, an unsupervised hierarchical clustering was applied to the whole DEG list to highlight gene expression pattern between sexes, and the heatmap was generated using ComplexHeatmap [53] R package. The gene ontology (GO) and Kyoto Encyclopedia of Genes and Genomes (KEGG) pathway enrichments of sex-biased genes were then performed using clusterProfiler (v 4.12.6) [54] R package.

### Functional analysis of sex-biased regulators

To identify the sex-biased transcription factors (TF), we downloaded the bovine TF and cofactor list from AnimalTFDB (v 4.0) [36], and overlapped them with the DEGs. The expression pattern of RNA-binding proteins (RBPs) was assessed in bovine blastocysts through retrieving a comprehensive list of 1542 manually curated RBPs from human research [55] and converting them into bovine orthologs, and the sex-biased RBPs were obtained by overlapping with DEGs. We also checked the protein–protein interaction (PPI) networks for the DEGs using the Search Tool for the Retrieval of Interacting Genes (STRING, v12.0, https://string-db.org/), and proteins with interaction score < 0.4 were removed from our results. The PPI network was visualized with the Python hiveplotlib module to show the interaction patterns.

To check if the DEGs were significantly enriched in specific gene groups or genomic regions, we created six gene groups, including protein coding genes, TF, TF cofactor, and genes located on autosome, X and Y chromosomes. Fisher exact test was used to determine if the sex-biased genes were significantly more or less enriched in specific gene groups. In addition, we defined the promoter regions as ± 1 kb around the transcription start site (TSS) and performed motif searching using homer [56] to predict the upstream regulators of the DEGs. The motif results were summarized and clustered based motif sequence similarity using JunJunZai R package [57].

### Dosage compensation analysis

To evaluate the relationship between DEGs and dosage compensation in sex-biased early embryo development, the X chromosome to autosome (X:A) expression ratio was calculated for female and male embryos. The pairwiseCI R package was used to get a 95% confidence interval for the ratio of median of X to the median of A as in previous research [58]. 1000 bootstrap replicates were included in the analysis where sampling from the original data was done with replacement and stratified by the group variables. Moreover, the X:A ratio was calculated for three gene categories, including expressed genes (TPM > 1), DEGs, and non-DEGs between sexes. To remove the effects of non-unique mapped reads on gene expression, we further excluded the paralog genes in the bovine genome and calculated the X:A ratio for each of the defined gene categories. We also checked the distribution of DEGs on the X chromosome, and the gene density was calculated in a 1 Mb window, which was visualized using RIdeogram [59] R package.

### Sex-biased alternative splicing event identification

To characterize the alternative splicing patterns in bovine early embryos, we followed the official protocol of rMATS-turbo [60] to obtain the sex-biased alternative events, including skipped exon (SE), alternative 5′ splice sites (A5SS), alternative 3′ splice sites (A3SS), mutually exclusive exons (MXE), and retained intron (RI). Briefly, rMATS-turbo utilized the widely used percentage spliced in (PSI) metric to quantify alternative splicing, which represents the percentage of transcripts that include a specific exon or splice site, as calculated from RNA-seq read counts supporting specific exons or splice junctions, normalized by the effective lengths of distinct transcript isoforms, then a generalized linear mixed model was applied to identify differential alternative splicing events between two groups. The statistically significant events were further selected with the following criteria: average RNA-seq read count > = 10 in both sample groups, filtering out events with average PSI value ranging from 0.05 to 0.95 in both sample groups, false discovery rate (FDR) < = 0.01, and between-group PSI value difference |ΔPSI|≥ 0.05. To examine potential regulatory interaction between differentially expressed splicing factors and sex-biased SE events, we used rMATS-derived SE events and junction reads data as input for rMAPS2 [61]. The motif mapping tool in rMAPS2 was run with default parameters and the bovine reference genome to predict the enrichment of FMR1 and HNRNPH2 binding motifs relative to SE events [62]. The functional enrichment of genes associated with differential alternative splicing (DSGs) was performed using clusterProfiler. Additionally, overlapping analysis was performed between DEGs and DSGs.

### Relationships between alternative splicing and known protein-coding domains

To explore if the differential alternative splicing (AS) events were associated with a switch between protein-coding isoforms and check if the SE, which was the most abundant AS event type in our analysis, can disrupt known protein domains, similar methods in a previous study were used [63]. Firstly, the coding sequence (CDS) was extracted from each isoform in the reference genome annotation and translated into corresponding amino acid sequence. Next, the known protein-coding domains were obtained from the PFAM database [64] and the amino sequences were used to query the PFAM database for selecting high-confidence amino acid alignment to determine protein-coding domains in each isoform. The high-confidence protein-coding domain alignments were defined with the following criteria: domain score > 10, domain E-value < 0.01, sequence alignment E-value < 1e-5, accuracy (hmmscan metrics) > = 0.8, and the proportion of the sequence domain aligned > = 0.9. To establish the relationships between SE events and protein-coding domains, the protein-coding domain locations were translated to genomic coordinates. Finally, the protein-coding domains were determined to be affected if they were overlapped with skipped exons, and enrichment analysis was conducted for the genes containing the affected protein-coding domains to check the potential functional changes.

### Long-read sequencing and differential isoform expression analysis

To capture the embryonic isoform diversity, pooled (n = 25) 4-cell embryos were analyzed using PacBio long-read transcriptome sequencing. Briefly, 4-cell embryos were collected at 44 h post insemination (hpi). RNA was extracted using PicoPure^™^ RNA isolation kit (KIT0204, Applied Biosytems^™^) with DNase treatment (RNase-Free DNase Set, Qiagen), ISO-Seq^®^ express 2.0 kit for cDNA synthesis, SMRTbell^®^ prep kit 3.0 for library preparation, and HiFi sequencing on SMRT Cell of a Revio platform (Genomics Resources Core Facility at Weill Cornell).

Long-read sequencing data were processed using the Iso-Seq pipeline [65]. Briefly, the high quality HiFi reads (predicted accuracy ≥ Q20) were processed with lima to conduct primer removal, and cDNA barcode identification. The poly(A) tail trimming and concatemer removal were conducted with isoseq refine. Then the isoform consensus clustering analysis was performed using isoseq cluster2, and the output isoforms were aligned to the bovine reference genome using pbmm2 [66]. Unique isoforms were obtained using isoseq collapse and 5′ degraded isoforms were removed using cDNA_Cupcake [67]. Finally, SQANTI3 (v 5.2.1) was used to classify isoforms with default parameters.

Next, we added the novel isoforms identified by long-read sequencing data to the cow reference genome. Using this new annotation, isoform expression in cow blastocysts was quantified using Salmon [68] together with the short-read RNA-seq data, and significantly differential expressed isoforms between sexes were identified using the swish method of fishpond [69] with q-value < 0.05. Additionally, the sex-biased exon usages (padj < 0.05 and log2FC > 2) were examined using DEXSeq [70] with default settings.

### Validation of differential expression genes

Real-time quantitative polymerase chain reaction (RT-qPCR) was used to validate the result of DEG identified by RNA-seq. 10 sex-biased DEGs were selected based on their fold change, p-value, and chromosome location. Five were upregulated in females (3 on the X chromosome and 2 on autosomes) and five were upregulated in males (3 on the Y chromosome and 2 on autosomes). Primer sequences were listed in Table S2.

A 10µl qPCR reaction contained 2 ng of cDNA, iTaq^™^ Universal SYBR^®^ Green Supermix (Bio-Rad), and 0.5 µM of primers. The qPCR conditions were: 95 °C for 3 min; 30 cycles of 30 s at 95 °C, 58 °C for 30 s, and 72 °C for 45 s; and 72 °C for 10 min. Relative expression was calculated using the 2^−ΔΔCT^ method with *GAPDH* and *H2AZF* used as reference genes. For evaluating the relationships between qPCR and RNA-seq results, we fit a linear regression model based on the relative expression fold changes derived from these two methods for the selected genes.

### Validation of alternative splicing events

Seven genes that with differentially exon skipping events between sexes were validated by PCR. A 10 µL of reaction contains 4 ng of cDNA, 5 µL of One*Taq*^®^ Quick-Load^®^ 2 × Master Mix (M0486L, New England Biolabs), and 5 pmol each of forward and reverse primers. PCR was conducted with the condition as follows: 15 min at 95 °C; followed by 35 cycles of 30 s at 95 °C, 30 s at 58 °C, and 45 s at 72 °C; lastly 72 °C for 10 min. PCR products were visualized by gel electrophoresis using a 2% agarose-TAE gel. Primers were designed to span the predicted skipping exon and were listed in Table S3.

## Results

### Sex-specific development paces

On Day-7 (168 hpi), expanded and hatched blastocysts were collected, while non-expanded blastocysts were kept in the culture medium for another 24 h. On Day 8 (192 hpi), non-expanded, expanded, and hatched blastocysts were all collected. Expanded blastocysts were further divided into two even halves for sex determination or transcriptomic sequencing. Each half contained both the inner cell mass and trophectoderm (Fig. 1A). In total, 444 blastocysts were collected. 412 of them were successfully sexed by the genomic PCR with sex-related primers and visualized on electrophoresis gel imaging (Fig. 1B**)**. A significant male-biased development was observed among the collected blastocysts, except for Day 7 hatched embryos (Table S4). On Day 7, 107 out of 155 expanded blastocysts were males (sex ratio = 2.23, p < 2.15e-06), whereas no significant sex ratio difference in hatched blastocysts due to few embryos hatched on Day 7 (8 males vs. 4 females, sex ratio = 2, p = 0.25). On Day 8, all developmental stages exhibited a significant male-biased development, including non-expanded (80/99, sex ratio = 4.21, p < 8.75e-10), expanded (91/112, sex ratio = 4.33, p < 3.73e-11), and hatched blastocysts (29/34, sex ratio = 5.80, p < 3.86e-05). These results aligned with the previous studies reporting a higher proportion of male expanded and hatched in vitro produced blastocysts on Days 7 and 8 in cattle [1, 13, 71, 72]. Moreover, male-specific genes on the Y-chromosome showed significant low/no expression in female samples, confirming a high accuracy of sex identification (Fig. 1C).

### Summary statistics of the RNA-seq data

Five male RNA-seq samples (5 pools of 2 half-cut male expanded blastocysts) and 6 female RNA-seq samples (6 pools of 2 half-cut female expanded blastocysts) with known sex information determined by PCR were used for RNA-seq. RNA-seq was performed using the Illumina PE150 platform. Data quality and mapping information were summarized in Table S4. A total of 399,685,192 raw reads were generated in the 11 samples, averaging 36,335,017 reads per sample with an error rate of 0.04%. GC content of the samples ranged from 45.23% to 47.60%, while sequencing quality metrics Q20 and Q30 were above 89.94% and 83.16%, respectively. Unique read mapping rates to the bovine reference genome averaged 80.94%, indicating robust data quality for bioinformatic analysis.

### Identification of sex-specific differentially expressed genes (DEGs)

**Fig. 2:**
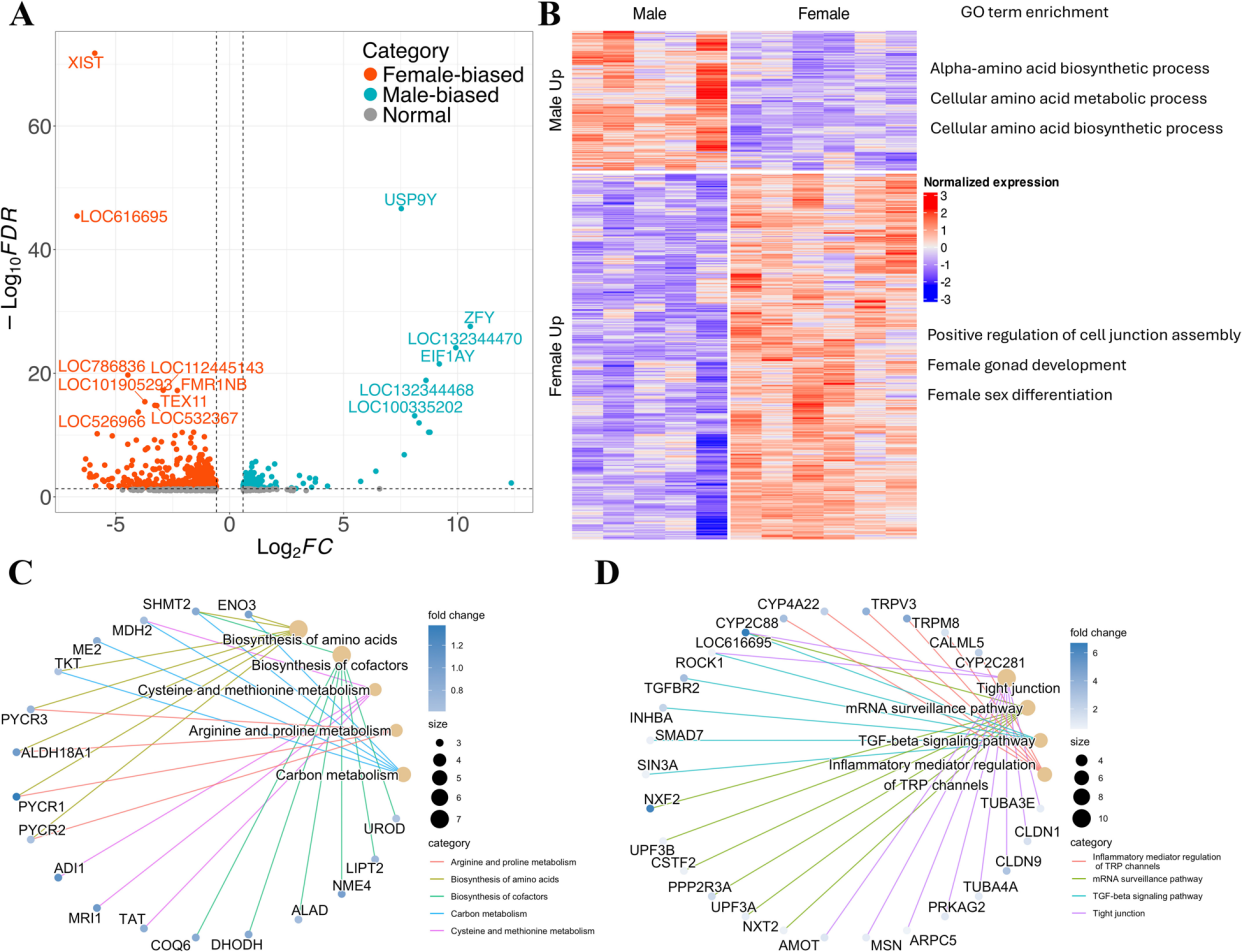
Sex-biased genes of bovine expanded blastocysts and functional enrichment. **A** Volcano plot showing all DEGs between male and female expanded blastocysts (BL). **B** Heatmap of sex-biased DEGs with associated GO functional terms. Color represents the z-score of normalized gene expression. **C** KEGG pathways enrichment of male-biased DEGs. Dot size indicates the number of genes associated with each pathway, and color represents fold enrichment based on the DESeq2 analysis between males and females. **D** KEGG pathways enrichment of female-biased DEGs. Dot size indicates the number of genes associated with each pathway, and color represents fold enrichment based on the DESeq2 analysis between males and females

To assess transcriptomic variation and clustering between sexes, principal component analysis (PCA) was conducted. Using the top 500 most variable genes, samples grouped into two distinct clusters, consistent with our experimental design, confirming that female and male embryos have different transcriptomic profiles at the early embryo stage (Figure S1). Sex-specific transcriptomic profiles were expected due to the different sex chromosome compositions between males and females. Indeed, 837 DEGs were identified between the two sexes, with 231 upregulated DEGs and 606 downregulated DEGs in male expanded blastocysts when compared to female expanded blastocysts (Fig. 2A, Table S5). Additionally, protein–protein interaction analysis revealed intensive interactions both within the DEG groups and across the non-DEGs (Figure S2), which may help to establish the sex-specific regulatory networks during early embryo development [35]. The two most differentially expressed genes *XIST* and *USP9Y*, located at the X chromosome and Y chromosome, were significantly upregulated in female and male expanded blastocysts, respectively. Among all the DEGs, 574 were located at the autosomes, 250 were located at the X chromosome, and 13 were located at the Y chromosome (Table S6).

### Function and regulation of sex-biased DEGs

To further explore these sex-specific transcriptomic differences, we performed an unsupervised hierarchical clustering of the entire sex-biased DEG list (Table S5). The DEGs were grouped into two clusters, corresponding to female- or male-biased genes (Fig. 2B). Gene ontology (GO) analysis showed that male-biased DEGs were primarily associated with metabolic processes, with top functions such as alpha-amino acid biosynthetic, cellular amino acid metabolic, and cellular amino acid biosynthetic process. In contrast, female-biased DEGs were enriched in processes such as female gamete generation, ovulation cycle process, positive regulation of cell junction assembly, and female gonad development (Fig. 2B). Moreover, the enrichment results of KEGG analysis showed that male-biased DEGs were enriched in metabolism and biosynthesis pathways, while female-biased DEGs were associated with inflammatory responses, cellular activity, TGF-beta signaling, and tight junction (Fig. 2C and D).

**Fig. 3:**
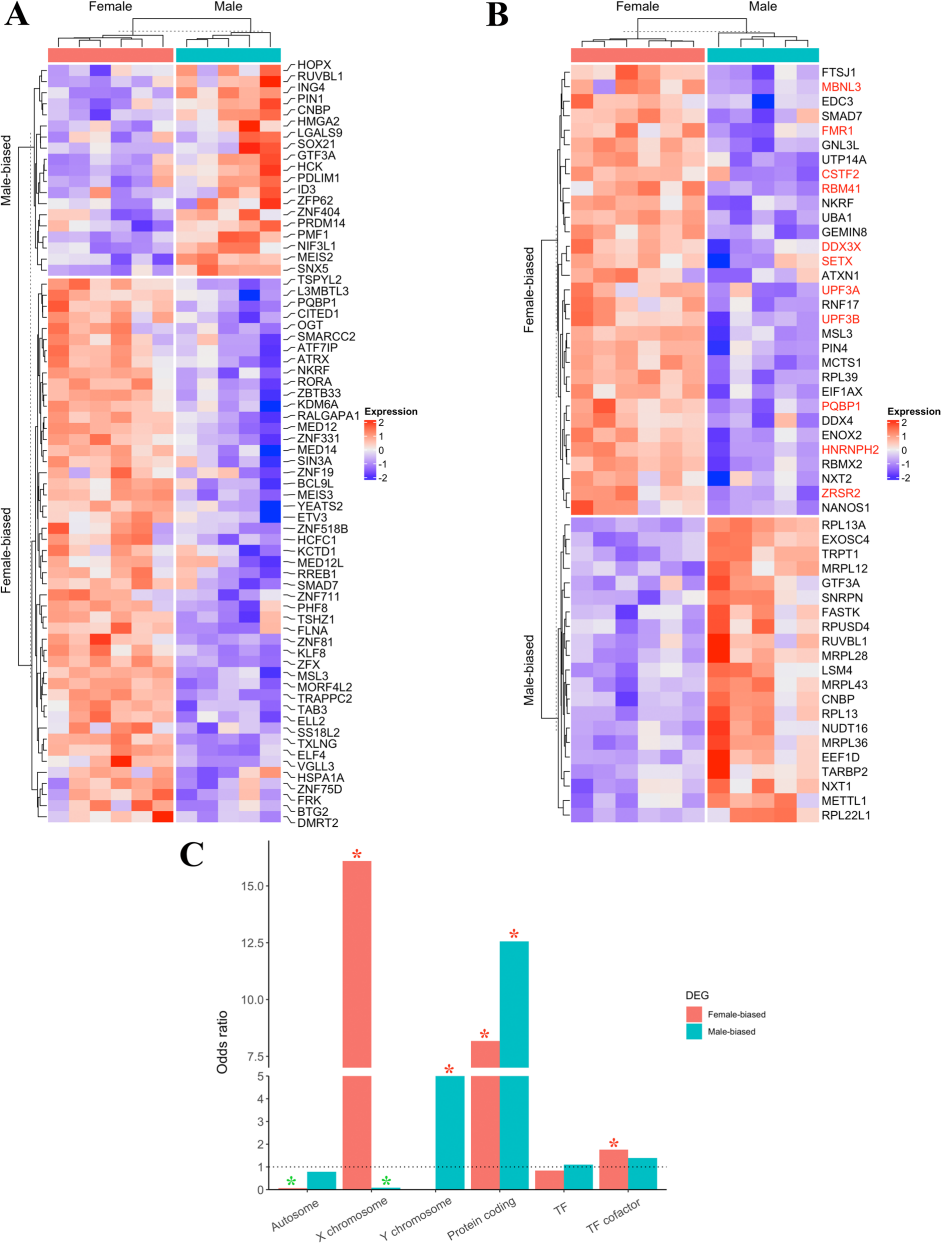
Heatmaps of Sex-biased transcription regulators and genomic distribution of DEGs. **A** Sex-biased transcription factors and cofactors. The color in the heatmap represents the z-score of normalized gene expression. **B** Sex-biased RNA binding proteins (RNA splicing factors highlighted in red). The color in the heatmap represents the z-score of normalized gene expression. **C** Enrichment analysis of DEGs in different gene groups. Green asterisks mean significantly less enrichment (p-value < 0.01) and red asterisks means significantly over enrichment (p-value < 0.01)

Examination of bovine known transcription regulators in sex-biased DEGs identified 68 sex-biased expressed TFs and cofactors, with 19 showing higher expression in males and 49 in female embryos, respectively (Fig. 3A). Specifically, we observed a few male-biased pluripotency regulators, including *HMGA2, SOX21,* and *PRDM14*. Moreover, we converted the human RNA binding proteins (RBP) list to cow orthologs and identified 31 and 21 RBPs exhibiting female- and male-biased expression, respectively (Fig. 3B**).** For example, FMR1 and HNRNPH2 are two known splicing factors annotated in the SpliceAid-F database [73], and they were significantly upregulated in female embryos compared to male embryos. Next, we determined the genomic distribution of sex-biased genes, we found that female-biased genes were significantly enriched in protein-coding genes, TF cofactors, and X chromosomes, while male-biased genes were significantly enriched in protein-coding genes and Y chromosome (Fig. 3C), indicating the distinct regulation of sex chromosomes in sex-specific early development.

Next, we performed de novo TF identification by motif calling based on promoter regions of the sex-biased DEGs. This analysis revealed several sex-biased motifs with predicted upstream binding TFs (Figure S3). For example, the female-biased motifs were dominated by TFs such as *ETV, ELK, ELF, SP*, and *KLF* families, whereas the male-biased motifs included *KLF, SP, ETS,* and *ELF* TF families. Shared TF families between the two sexes included *PABPC1, NFY, ELK4, ELFs, SPs,* and *KLFs,* with *PABPC1* showing the most significant enrichment (Figure S3)*.*

### X-chromosome dosage compensation in sex-specific embryos

**Fig. 4:**
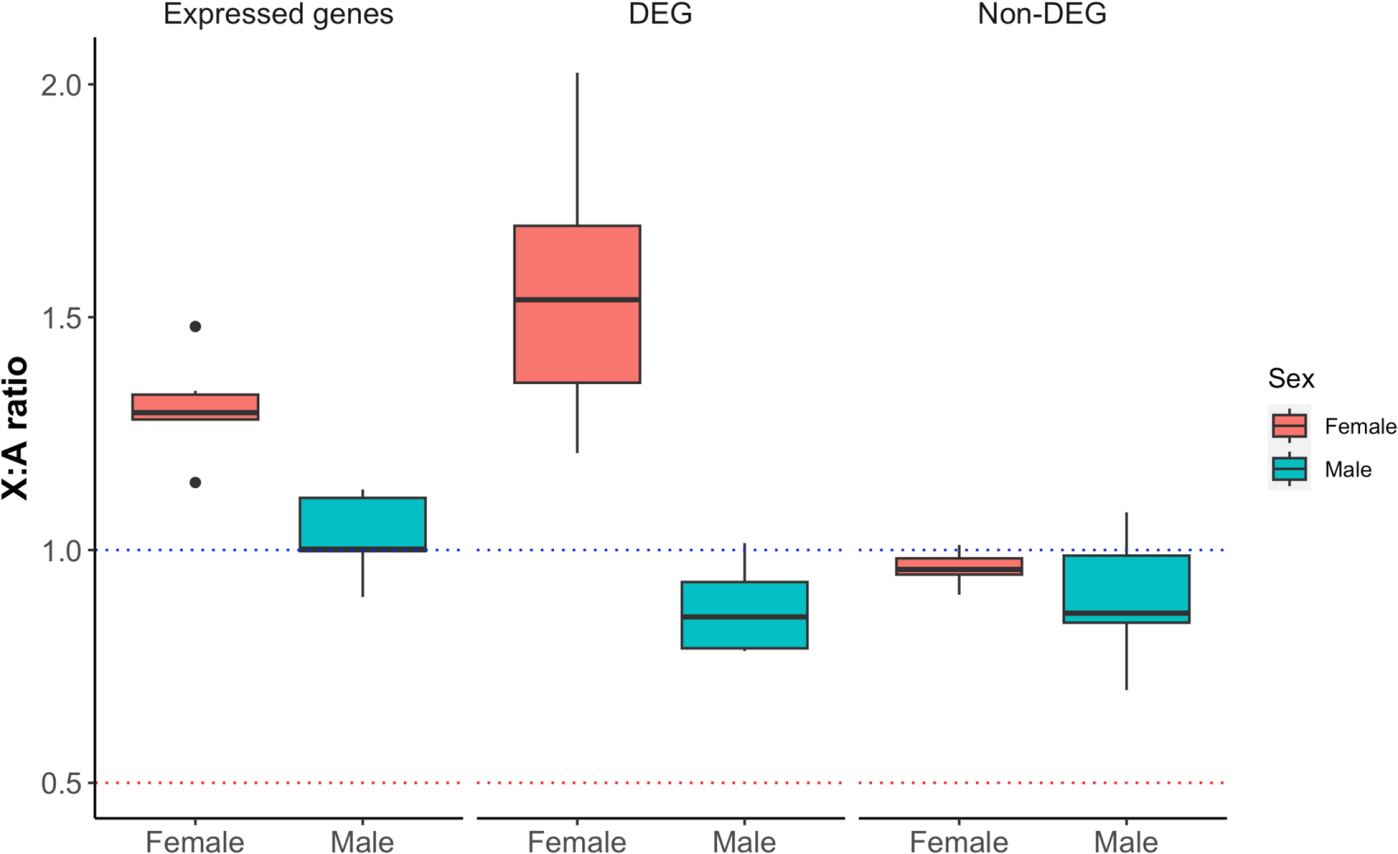
Gene dosage compensation. The X:A expression ratio was calculated using expressed genes (TPM > 1), DEGs and non-DEGs in female and male expanded blastocysts

To investigate the X-chromosome dosage compensation at the blastocyst stage, X:A ratios were calculated in three categories: expressed genes (TPM > 1), DEGs, and non-DEGs for both sexes (Fig. 4). An X:A ratio of 1.0 or higher indicates complete compensatory upregulation of X-linked genes compared to autosomes, 0.5 suggests no compensation, and values between 0.5 and 1 reflect partial compensation [74]. In male embryos, the X:A ratios were consistently around 1 in expressed genes and slightly lower than 1 in male-biased DEGs (Fig. 4), indicating a complete and incomplete dosage compensation, respectively. In contrast, the X:A ratios varied in female embryos, with the highest value (> 1.5) observed in female-biased DEGs, around 1.3 for the expressed genes, and close to 1 for non-DEG. These findings suggested that the dosage compensation levels differed among different subgroups of the X-linked genes, with female-biased X-linked DEGs being the primary drive of dosage.

Given the fact that the X chromosome is abundant with paralog genes in mammals [75, 76], which might affect the quantification of the X-linked genes, we recalculated the X:A ratio after excluding the paralog genes in the bovine genome. Interestingly, the X:A ratio was reduced when excluding the paralog genes, especially in the sex-biased DEGs group, further supporting the enrichment of paralog genes on the X chromosome contribute to the dosage compensation (Figure S4).

To further assess if the elevated expression of X-linked DEGs in females was associated with X chromosome inactivation (XCI) and located to specific XCI regulatory regions such as the X inactivation center, XCI escapes, gene-rich areas, or pseudoautosomal regions (PAR), which is known to escape XCI [75, 77]. We collected a total of 52 known XCI escapee candidates from previous research [78], and compared their genomic distribution with the female-biased X-linked DEGs and all annotated X-linked genes (Figure S5). The result revealed similar distribution patterns between female-biased X-linked DEGs and XCI escapee candidates, both concentrated in certain gene-rich regions along the X chromosome. Particularly, 17 of the 52 XCI escapee candidates were exhibiting significantly female-biased expression on the X chromosome (Table S6). However, these female-biased X-linked DEGs did not overlap with known PAR regions, which were located at the distal end of the bovine X chromosome [79]. Overall, our analysis suggests the upregulated expression of female-biased DEGs was partly due to the escapee gene clusters, while not linked to specific XCI regulatory regions or PAR regions of the X chromosome.

### Alternative splicing in sex-specific embryos

**Fig. 5:**
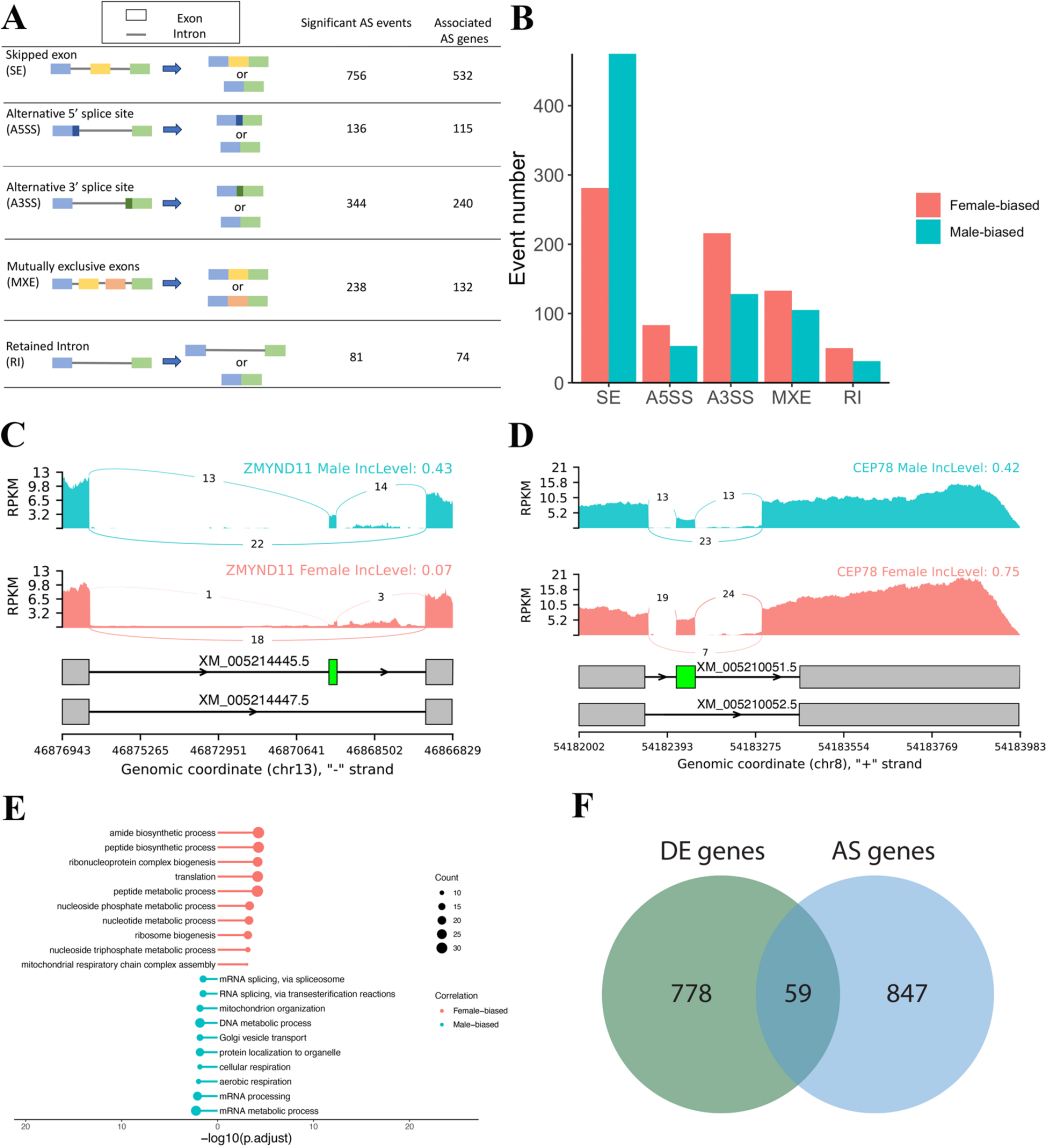
Sex-biased alternative splicing events in bovine expanded blastocysts. **A** Schematic of five categories of alternative splicing (AS) events identified by rMATs, with numbers of sex-biased differential AS events (right panel) passing threshold (FDR < = 0.01 & delta PSI value ≥ 0.05), and AS-associated gene count. **B** Event number of female-biased and male-biased differential AS events. **C** Example of a male-biased SE event in ZMYDN11, showing a higher inclusion of the transcripts that contain the skipped exon in male blastocysts. Numbers on the arcs indicated the read counts spanning splicing junctions. Gray boxes at the bottom panel represent constitutive exons, and the green box indicates the alternative exon. **D** Example of a female-biased SE event in CEP78, showing a higher inclusion level of the transcripts that contain the skipped exon in female blastocysts. Numbers on the arcs indicated the read counts spanning splicing junctions. Gray boxes at the bottom panel represent constitutive exons, and the green box indicates the alternative exon. **E** GO term enrichment of all genes associated with sex-biased AS events. **F** Venn diagram showing overlapped genes between differentially expressed gene list and genes associated with sex-biased AS event

Next, we evaluated the contribution of alternative splicing to sex-specific early embryonic development. Using rMATs, we identified 27,875 AS events, with 1555 showing significantly sex-biased difference (FDR < = 0.01; delta PSI value ≥ 0.05) across five different event types: skipped exon (SE), alternative 5’ splice sites (A5SS), alternative 3’ splice sites (A3SS), mutually exclusive exons (MXE), and retained intron (RI) (Fig. 5A, Table S7). Among the five types, SE event was the most dominant AS category between sexes (Fig. 5B**)**, with 281 SE events being female-biased and 475 SE events being male-biased. For example, *ZMYND11*, a gene involved in epigenetic regulation [80], exhibited significant high exon inclusion in male embryos (Fig. 5C), while *CEP78* showed a significantly high exon inclusion level in female embryos (Fig. 5D)**.** Then, to investigate whether the sex-biased SE events could be regulated by these two female-biased splicing factors, we analyzed the binding potential of FMR1 and HNRNPH2 using rMAPS2. In female-biased SE events, FMR1 showed significantly increased binding potential downstream of the alternative exons, whereas in male-biased SE events, HNRNPH2 exhibited significantly increased binding potential upstream of the alternative exons, suggesting distinct regulatory roles in sex-specific alternative splicing (Figure S6).

Furthermore, functional enrichment analysis of sex-biased AS events associated genes revealed that the female-biased AS genes were enriched in amide and peptide biosynthetic processes, while the male-biased AS genes were enriched in mRNA processing and metabolic processes (Fig. 5E). Moreover, overlapping analysis between sex-biased AS genes and sex-biased DEGs identified 59 AS genes that also exhibited significant differential expression at the gene level (Fig. 5F). GO analysis of these overlapping genes revealed their association with translation regulation, protein metabolic process, mitochondrial respiratory chain, and oxidoreductase activity, all of which played essential roles in early embryogenesis, indicating their potential roles in sex-specific developmental regulation (Table S6).

Finally, to determine whether alternative exon usage overlaps with protein-coding domains and contributes to protein diversity, we mapped the known protein domains to sex-biased SE events. A total of 280 and 438 skipped protein domains were identified in female and male embryos, respectively (Table S8). Functional enrichment analysis of associated genes exhibited skipped protein domains revealed that energy metabolism and protein process were enriched in female embryos, while the transport and catabolism process were enriched in male embryos (Table S8).

### Differential isoform level expression in sex-specific embryos

Isoform diversity is a key mechanism by which the genome generates transcriptome complexity during early development. To achieve a more complete annotation of isoforms in early embryos, we used long-read sequencing, which enables direct sequencing of the full-length transcripts with low error rates. Long-read data were generated from pooled 4-cell stage bovine embryos, allowing us to identify the novel isoforms and splicing variants relevant to preimplantation development. As a result, we identified 286,711 unique isoforms that mapped to 14,084 novel genes and 14,353 annotated genes. Through comparison with the cow reference genome, the long-read isoforms were classified into different categories, including full splice match (FSM), incomplete splice match (ISM), novel in catalog (NIC), novel not in catalog (NNC), intergenic, antisense, fusion and genic genomic (Figure S7A). In total, we obtained more than 140,000 novel isoforms (NIC and NNC categories) from the early embryos (Figure S7B). To assess the isoform features, we found more than 50% of the genes only have one isoform, around 10% of the genes have 2–3 isoforms, and the rest of the genes have at least four isoforms (Figure S7C). Additionally, more than 90% of the isoforms contained the canonical splicing sites and had relatively high short-read coverage associated with the splice junctions, suggesting good quality of the long-read data (Figure S7D).

**Fig. 6:**
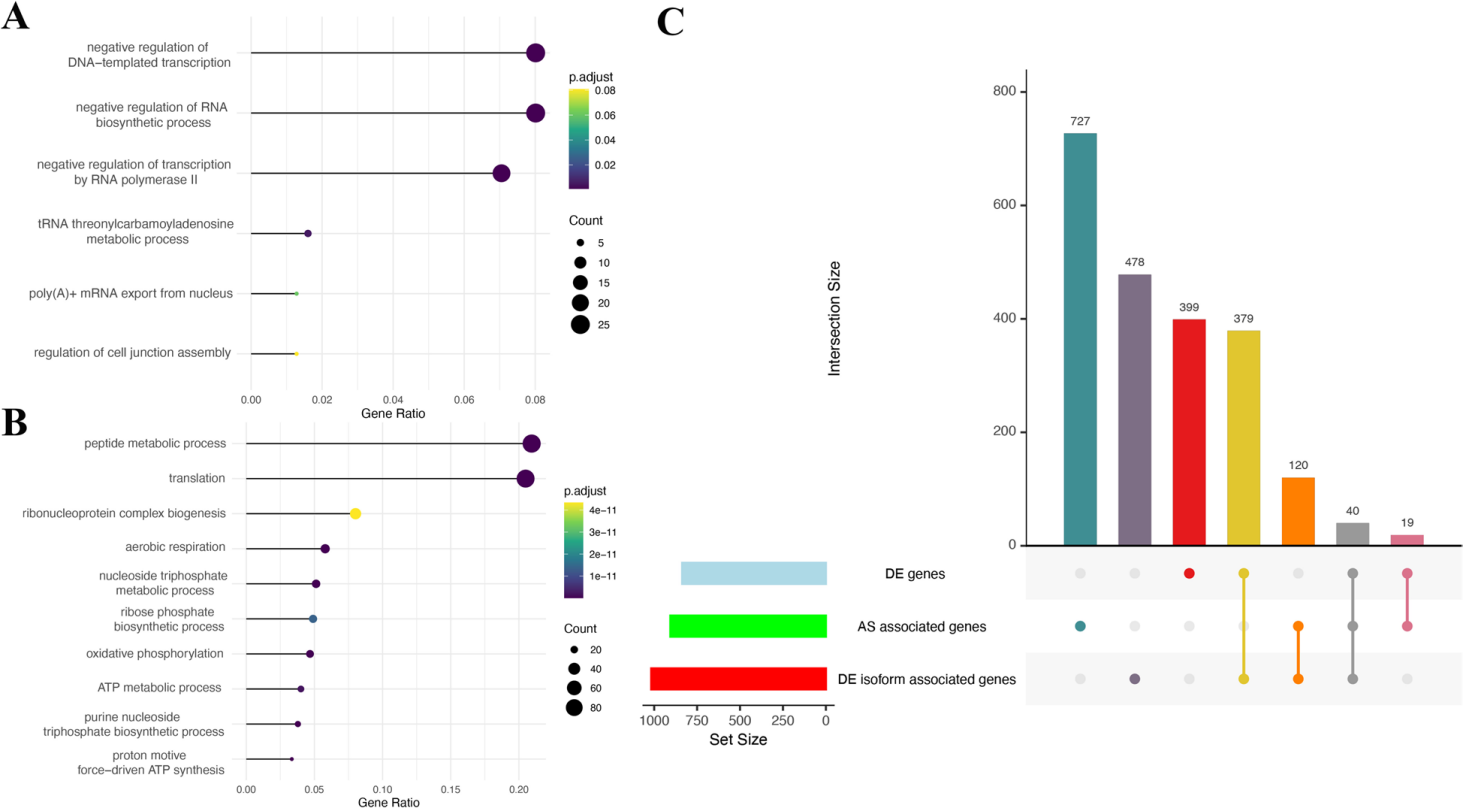
Functional enrichment of sex-biased isoforms and associated genes. **A** GO functional enrichment of female-biased isoforms. **B** GO functional enrichment of male-biased isoforms. **C** UpSet plot showing overlap genes among DEGs, sex-biased AS-associated genes, and sex-biased isoform-associated genes. The horizontal bars on the left represent the total number (set size) of genes in each category. The vertical bars on the right represent the number (interaction size) of genes in the interactions between categories, as indicated by the connected colored dots at the bottom

To integrate long-read and short-read transcriptomic data, we first incorporated the novel isoforms identified from long-read sequencing of 4-cell stage embryos into the bovine reference genome to generate an updated transcript annotation. Using this enhanced reference, we quantified isoform expression in expanded blastocysts using short-read RNA-seq data from male and female embryos, and the different isoform analysis was then performed to identify sex-biased isoforms. A total of 1151 differentially expressed isoforms were obtained in this study, including 514 female-biased isoforms and 637 male-biased isoforms, which were associated with 1017 genes (Table S9). According to the functional enrichment of the differential isoform-associated genes, we showed that negative regulation of DNA-templated transcription and RNA biosynthesis were enriched in female embryos (Fig. 6A), and the peptide metabolic processes were enriched in male embryos (Fig. 6B), aligning with the slow development pace observed in female embryos. An UpSet plot was used to visualize gene overlaps among DEGs, sex-biased AS-associated genes, and sex-biased isoform-associated genes (Fig. 6C). We identified 419 genes that were both DEGs and had differentially expressed isoforms, of which 379 were shared exclusively between DE genes and DE isoform-associated genes, and 40 were shared among all three categories. We also identified 478 genes that only contained differentially expressed isoforms (Fig. 6C, Table S10). Additionally, we observed 160 genes shared between sex-biased AS events and DE isoforms-associated genes, including 120 exclusives to those two categories and 40 overlapped with all three. Notably, only 59 overlap genes between sex-biased AS events and DEGs, of which 19 were exclusive to these two categories, suggesting that sex-biased AS events contribute more to differential isoform expression than to gene-level sex-biased DEGs (Fig. 6C). For example, *APOE* showed consistent sex-biased expression at both genes and associated isoforms. *KDM6A* was sex-biased at the gene level without significant difference at the isoform level. *GOLM2* showed no significant difference at the gene level, but contained one isoform that is significantly male-biased, highlighting the added resolution gained from isoform-level analysis (Figure S8). Furthermore, analysis of differential exon usage between sexes revealed 7 exons exhibit biased usage in female or male embryos (padj < 0.05 and log2FC > 2). These exons were associated with 5 genes, including *DENND1B*, *DIS3L2*, *DOCK11*, *IL1RAPL2*, and *ZRSR2Y* (Figure S9 and Table S11), which may have sex-specific regulation in early embryo development.

### Validation of sex-biased DEGs and alternative splicing events

**Fig. 7:**
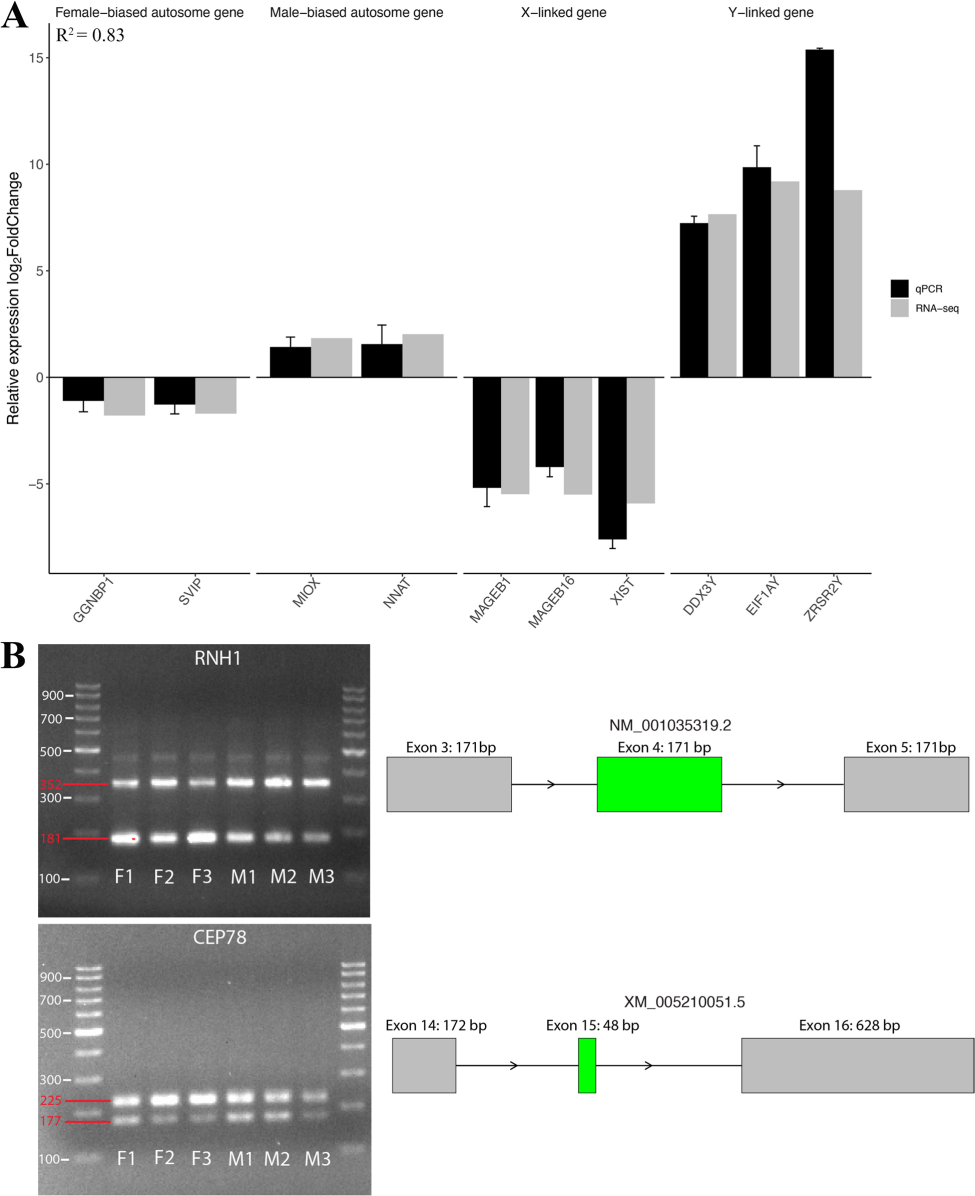
Validation of DEGs and AS events. **A** Validation of DEGs with qPCR. **B** Agarose gel of two selected alternative splicing associated genes (RNH1 and CEP78). Primers were designed to span the predicted skipping exon. The upper band contains the transcripts that include the skipped exon, while the lower band contains the transcripts that do not include the skipped exon. F1, F2, and F3 are female embryos; while M1, M2, and M3 are male embryos

For validating the gene expression pattern between sexes from RNA-seq, we selected the top sex-biased genes (log2FC > 1.5) and quantified their expression using PCR methods, including female-biased autosomal genes (*GGNBP1*, *SVIP*), male-biased autosomal genes (*MIOX*, *NNAT*), female-biased X-linked genes (*MAGEB1*, *MAGEB16*, and *XIST*), and male-biased Y-linked genes (*DDX3Y*, *EIF1AY*, and *ZRSR2Y*). The relative expression fold change among all examined genes exhibited similar trends between RNA-seq and PCR results (R^2^ = 0.83, Fig. 7A), indicating the robustness of our RNA-seq analysis.

To validate the sex-biased AS events, we selected five significantly differential SE events and designed specific primers to amplify the target regions of the alternative exons, including one female-biased SE in *CEP78* and four male-biased SEs in *RNH1*, *CEP350*, *ZMYND11*, and *WWC2*, respectively **(**Figs. 7B and S10). In this study, we mainly focused on SE events because they were the most abundant sex-biased alternative splicing events. Moreover, considering the defined structure and flanking junctions between the transcripts that having the AS event, SE events were chosen to validated because their size difference is able to be recognized from the agarose gel after running the PCR products. Gel electrophoresis results showed that in male embryos, the exon inclusion band (352 bp) of *RNH1* was brighter than the exon-skipped band (181 bp), while in female embryos, the exon inclusion band (225 bp) of *CEP78* was more abundant than the exon-skipped band (177 bp), consistent with the sex-biased exon inclusion level observed in RNA-seq data (Fig. 7B). Further confirmation showed that the observed PCR product length differences were due to the skipped exons, and the exon inclusion level calculated from PCR gel electrophoresis aligned with the RNA-seq calculated results (Figure S11).

## Discussion

Sex differences are a widely studied topic in developmental, nutritional, and medical research, as the distinct composition of sex chromosomes influences hormone synthesis, brain function, immune regulation, and body structures, causing males and females to respond differently to the same stimuli. While many of the differences become apparent only after the formation of sex glands and the onset of hormone production, embryos already exhibit sex-specific differences during the preimplantation period, even before sex-specific hormonal regulation [21].

In this study, we investigated the transcriptomic differences between male and female bovine embryos in early development. Using short-reads RNA-seq and long-reads ISO-seq, we identified sex-biased DEGs, AS events, and isoform-level expression patterns. Male embryos exhibited faster development, driven by upregulated metabolic pathways, while female embryos showed slower development associated with negative regulation of transcription and RNA biosynthesis. Additionally, distinct X-chromosome dosage compensation was observed, with an X:A ratio greater than 1 in female embryos, suggesting both X-chromosome upregulation (XCU) and incomplete X-chromosome inactivation (XCI). Overall, these findings provide new insights into the regulatory mechanisms underlying sex-specific embryogenesis.

### Sex-specific embryonic development in different species

Sex differences in early embryos, observed before gonadal formation and hormone synthesis, are well-documented across species. Previous studies in cattle have shown that in vivo developed male blastocysts retrieved from the cows exhibit faster development, higher proliferating cell numbers, and elevated expression of pluripotent genes [31]. Similarly, our study demonstrated that the in vitro produced bovine male blastocysts developed faster than female embryos, consistent with previous findings in IVF embryos in cattle [1, 13, 14, 17]. This phenomenon is conserved among other mammals, including humans [3–9], mice [10–12], pigs [15, 16], and sheep [17], as well as in porcine in vivo embryos [15]. However, the observations in humans are more complex. While some studies suggest no difference in live birth sex ratio following IVF embryo transfer [81], others have observed accelerated male development in ICSI-produced embryos but not in IVF embryos [22]. These discrepancies may arise due to variations of embryo origins, culture conditions, and study designs, as human studies often involve embryos from oocyte or sperm donors with fertility issues.

Interestingly, environmental factors such as the composition of in vitro culture medium and culture conditions also significantly influence sex-specific development. For example, glucose supplementation in the culture medium was found to accelerate male development and increase cell numbers in male bovine blastocysts [21, 22, 31]. Moreover, cultures supplemented with serum or plates coated with granulosa cell monolayer enhance the male-to-female ratio at Day 6 and Day 7, indicating faster cleavage in male embryos under these conditions [1]. In contrast, female embryos exhibit greater resilience and higher survival rate compared to males under heat stress, both in vivo and in vitro culture [82]. These studies highlighted the sex-specific genome regulation in driving the differences in development speed and response to different culture conditions. However, the underlying regulatory mechanisms remain incompletely understood.

### X dosage compensation in sex-biased embryogenesis

Transcriptional sexual dimorphism in early embryos is influenced by factors such as the expression of X/Y-linked genes, XCI escapees, and interactions between sex chromosomes and autosomal genes [21]. In bovine somatic tissues, studies have reported a complete X dosage compensation for “dosage-sensitive” (ubiquitously expressed) genes with X:A ratios around 1 and no sex difference [75]. In bovine in vivo embryos, X:A ratios vary dynamically: they start at 1 in maturated oocytes, decrease in zygotes with an inactive paternal X, rises to peak levels (X:A > 1) during ZGA and reduce to around 1 after the morula and blastocyst stages, where it stabilizes [75]. Interestingly, in IVF embryos, the X:A ratio showed distinct patterns compared to in vivo embryos with X:A ratio exceeding 1 in IVF blastocysts [75]. These findings reflect the intricate regulation of X dosage during bovine early embryo development and the influences of the embryonic development environment.

According to Ohno’s hypothesis of X dosage compensation [83, 84], in somatic cells, the XCU compensates for the silencing effects of XCI by upregulating genes on active X in females, maintaining an X:A ratio close to 1. Consistent with this, our study found that the X:A ratio for expressed genes was around 1.3, while for female-biased DEGs, it was 1.5 in female blastocysts. This upregulation of X-linked genes in female embryos is also consistent in human embryos [32, 33]. The elevated X:A ratio of the expressed and female-biased DEGs likely reflects the XCU coupled with an incomplete XCI, as *Xist* expression is initiated at the morula stage and may not yet be completely established in blastocysts. Specifically, the upregulation of female-biased DEGs may result from XCU on the active X chromosome (Xa) while some genes remain active on the Xi due to incomplete XCI, or they are classified as XCI escapees, contributing to the elevated X:A ratio. In this study, we found a small subset of female-biased DEGs were known XCI escapee candidates in the bovine genome, such as MED12 and SMARCA1. MED12 is a subunit of the mediator complex, and it plays a crucial role in embryonic development, particularly in regulating the Wnt/β-catenin signaling pathways of mouse embryos [85]. SMARCA1 is a member of the ISWI family of chromatin remodeling proteins, associated with gene expression regulation and cell lineage determination [86]. All these findings suggested that XCI escapees might promote the sex-biased development in bovine early embryos, which needed to be explored in future studies.

In mice, XCU has also been observed during early embryogenesis, and males achieve XCU upon ZGA while there are two waves in females: the first in response to imprinted XCI after ZGA and until blastocyst stage, then the second during random XCI at post-implantation stage [87, 88], both mediated by *Xist* [89, 90]. Impaired XCI leads to a subsequently skewed sex ratio of embryos with female-biased peri-implantation defects [91]. In mouse IVF embryos, this skewed sex ratio has been linked to reduced expression of XCI regulators, such as *Rnf12* or *Xist*, which can be rescued by *Rnf12* overexpression [91]. These findings suggest that the incomplete XCI and high expression of XCI escapees in female blastocysts may partially contribute to a slower development rate and the skewed sex ratio in early development. However, further studies are needed to validate this hypothesis.

### Sex-biased DEGs in early embryos

Cellular programming, activity, epigenetic modifications, and gene transcription are closely linked to metabolism, with metabolites signaling primarily influencing epigenetics and gene transcription, while metabolism pathways are largely regulated by post-translational mechanisms [92]. During oocyte maturation and early embryo development, maturation and initial cleavages rely primarily on maternal deposits, such as mitochondrial, mRNAs, proteins, and lipids, whereas later divisions depend more on embryonic synthesis and nutrients from the developmental environment [93–95]. Upon blastocyst formation, more oxygen, nutrients, and energy consumption are required for cell growth and division [93]. In drosophila embryogenesis, abnormal high dNTP levels induced by expression of feedback-insensitive ribonucleotide reductase accelerate nuclear cleavages while reducing zygotic transcription, resulting the failure in early gastrulation [95]. Additionally, nutrient depletion, such as glucose or phosphate removal from culture media, often leads to hamster or human embryo arrest in vitro [96–98]. The inadequacies of the in vitro culture condition compared to the in vivo environment contribute to delayed embryo development in hamsters [99]. These findings suggest that a more active metabolism correlates with faster early embryonic development.

In our study, male-biased DEGs were enriched in amino acid biosynthetic and metabolic processes (e.g. *ENO3*, *PYCRs*), mitochondrial structures (e.g. *COQ6*, *MRPL43*, *SHMT2*), and carbon metabolism (e.g. *SHMT2*, *ME2*, *TK*), suggesting a higher metabolic activity in male blastocyst. This finding is consistent with a microarray study, which reported high expression of genes related to metabolism, mitochondrial regulation, and cell cycle processes in male blastocysts compared to females [29]. Among amino acids crucial for cell growth and embryo development, proline enhances mitochondrial function and cell proliferation [100, 101], arginine promotes trophoblast development [102], and methionine, essential for DNA methylation, is linked to pluripotency maintenance [103]. Additionally, increased mitochondrial activity is associated with aster transcription, translation, and proliferation in human cells culture [104]. Therefore, enriched amino acid and carbon metabolism may accelerate blastomere cleavage in males, contributing to earlier development to the blastocyst stage.

In contrast, female-biased DEGs were associated with female-specific developmental processes, including gamete generation, gonad development, and ovulation cycles, with involvement in pathways such as mRNA surveillance, TGF-beta signaling, and inflammatory mediation. While gonad development is not initiated at this stage, the upregulation of female-specific development genes suggests a sex-specific developmental difference. Moreover, the mRNA surveillance pathway plays a critical role in detecting and degrading abnormal mRNA, and the disruptions in this pathway have been linked to lower embryo viability and infertility in mice [105]. Additionally, TGF-beta signaling, which regulates pluripotency in embryonic stem cells [106], is highly expressed at the 4- to 8-cell stages but declined at the blastocyst stages in cattle [107, 108]. Dysregulation of this pathway way leads to elevated TGF-beta signaling levels and results in bovine blastocysts degeneration [109, 110]. These findings suggested that the in vitro produced bovine female blastocysts exhibit female-specific regulatory mechanisms during early development, whereas male blastocysts show higher metabolic activity. However, a transcriptomic study has reported a higher metabolic activity in in vivo produced bovine female blastocysts [30], indicating that the developmental environment may contribute to this difference.

According to previous studies, faster cleavage can be associated with enhanced metabolic activity, but it does not always directly indicate better embryo quality. For example, in mice, faster-developed IVF embryos exhibit higher rates of imprinting errors, such as imprinted methylation loss of *H19* and *Snrpn* [111], whereas slower-developed embryos are more prone to development arrest. Both aberrantly developed embryos show higher aneuploidy rates compared to moderate cleavage rate IVF embryos, which more closely resemble the imprinted pattern of in vivo embryos, leading to lower implantation and pregnancy rates [112]. These findings indicate that faster-developing blastocysts are not necessarily of higher embryonic quality.

Beyond gene functional analysis, upstream transcriptional regulators of DEGs also exhibit sex-specific differences. Among the male-biased transcription factors, *HMGA2, SOX21*, and *PRDM14*, which are key regulators of pluripotency maintenance [113–115], align with enriched pluripotency pathways in males. Among the sex-biased RBPs, 11 RBPs were known to regulate alternative splicing (AS) in humans [116–122], including *MBNL3, FMR1, CSTF2, RBM41, DDX3X, SETX, UPF3A, UPF3B, PQBP1, HNRNPH2, and ZRSR2*. Notably, *FMR1* and *HNRNPH2* are two known splicing factors associated with fragile X syndrome and X-linked development disorders [123, 124], they exhibited significant female-biased expression. Moreover, the female-biased RBPs showed enrichment for AS regulation, supporting the observation that AS profiles are distinct between female and male blastocysts.

Besides overlapping DEGs with known bovine TF database, de novo motif calling enabled target-specific TF identification, revealing the most enriched sequence at DEG promoters. For example, *PABPC1,* also known as an embryonic poly(A)-binding protein, plays a key role in mRNA stabilization and translation initiation and is critical for preventing early embryonic arrest in human and mouse embryos [125]. Moreover, several TFs identified in promoters of sex-biased DEGs are critical to embryo development. For instance, *ETVs* (*ETV1, ETV5*), *ELK1*, *ELF1*, and *Sp1* are known to regulate the transcription of many genes during zygotic genomic activation (ZGA) [126–129]. The *KLF* family plays an important role in embryonic development and maintaining embryonic stem cell pluripotency [126, 129–131]. Interestingly, one common predicted motif from both sexes, *ELF4,* a key ZGA and epigenetic reprogramming regulator in pigs [132], was also identified as one of the female-biased DEGs in our analysis, suggesting that its expression also contributes to regulating its downstream genes. These results showed a more specific upstream regulation of sex-biased DEGs between male and female blastocysts.

### Sex-biased alternative splicing in early embryos

Alternative splicing plays a crucial role in transcriptome diversity by selectively including or excluding exons and introns from the same gene [133]. The resulting transcripts are often expressed in a developmental stage-specific manner and contribute to various biological processes, including gonadal differentiation [134], sex determination [135], and stress responses [136]. Importantly, AS helps in establishing the complex regulatory networks in mammals by generating multiple proteins from a single gene [137]. Sex-biased AS serves as an alternative mechanism for sexually dimorphic traits, complementing the sex-biased gene expression [138]. In Drosophila, sex-biased AS has been observed at the 0–2 h stage, with alternative first exon usage as the most common type [139].

In this study, we identified more than 1500 significant sex-biased AS events in bovine expanded blastocysts, with SE events being the most abundant. Specifically, male-biased SE events were more prevalent than those in females, which might due to that the increased activity of splicing regulators in female embryos could contribute to alternative exon exclusion. Analysis of protein domains affected by sex-biased SE events revealed distinct differences between male and female embryos. In male embryos, the most frequently skipped protein domains included FERM_N, which is involved in localizing proteins to the plasma membrane [140], HMG_box, which regulates DNA-dependent processes [141], and Nramp, which is associated with transmembrane transport [142]. In female embryos, the top skipped protein domains were DSPn, which plays a role in dephosphorylation [143], LRAT, which catalyzes vitamin A esterification [144], and Septin, which is essential for cytokinesis and other diverse cellular functions [145]. Moreover, functional enrichment analysis of all male-biased AS associated genes revealed significant correlations with mRNA metabolic process and mitochondrial functions, supporting the observation that energy metabolism was more active in male embryos. These findings highlight the potential role of AS in shaping sex-specific metabolic and developmental pathways during early embryogenesis and suggest that sex-biased AS events may lead to functional modifications in protein activity, potentially altering metabolic processes in female embryos.

Epigenetic modifications are known to control transcription and splicing outcomes. For example, histone modification regulates AS by recruiting splice factors, while DNA methylation primarily affects splicing by modulating RNA Pol II elongation rate or causing transcription pause [146]. Histone modification-associated AS events have been reported to be involved in embryonic stem cell fate decision, cell self-renewal, and pluripotency maintenance [147, 148]. Non-coding RNAs, including short (< 200 nt) and long (> 200 nt), also contribute to AS regulation. These RNAs can mediate the activity of the splice factor or directly interact with pre-mRNAs. Long non-coding RNAs (lncRNAs) have been reported to affect AS by forming RNA–DNA duplex or RNA-RNA hybrid, or by interfering with the recruitment of chromatin remodelers [149]. In addition, X/Y sperm-specific small RNAs have been found to be involved in regulating the catabolic processes [150]. Some sperm-borne microRNA persist from the zygote to the blastocyst stage and may influence early embryonic genome regulation [151]. This raises the possibility that sex-specific sperm small RNAs contribute to the observed AS profiles differences between female and male blastocysts. Another layer of epigenetic regulation involves RNA modifications, such as N6-methyladenosine (m6A), which can affect AS by interacting with splicing factors [152]. Despite these insights, the precise mechanisms of how these epigenetic modifications regulate sex specific AS in early embryos remain poorly understood. We believe future studies integrating epigenomic and transcriptomic approaches will be essential in defining these complex regulatory layers.

### Sex-biased isoform expression in early embryos

By incorporating novel isoforms identified from long-read sequencing, we obtained a more comprehensive view of bovine embryonic isoform diversity, identifying more than 1100 sex-biased isoforms that correspond to more than 1000 genes. Considering both gene-level expression and isoform dynamics provided deeper insight into how different genes were finely tuned in a sex-specific manner. For example, APOE encodes two isoforms that differ in apolipoprotein domain structure, but only one isoform was abundant in bovine blastocysts and exhibited significantly male-biased expression. Given APOE's known roles in lipid transport and neurodevelopment, this male-biased isoform may affect the early nervous system development or metabolic regulation during embryogenesis [153]. In contrast, KDM6A, a gene involved in chromatin remodeling and developmental gene regulation, produced 4 isoforms in bovine blastocysts. All KDM6A isoforms showed slightly higher expression in female embryos, contributing to an overall significant female-biased gene at the gene level. These findings are consistent with KDM6A's role in cell differentiation and epigenetic regulation during early development [154].

Importantly, this study identified hundreds of genes exhibiting sex-biased expression only at the isoform level, without significant differences at the gene level. These sex-biased isoforms may play key roles in sex-specific development pathways, yet they would have been missed by conventional gene-level analysis. For instance, GOLM2, which encodes a Golgi membrane protein and has two isoforms, showed comparable gene-level expression between sexes. However, one isoform was highly expressed in male embryos, suggesting that isoform-specific regulation of Golgi-associated processes may contribute to male-specific cellular organization or signaling during blastocyst development [155].

Examining the relationship between sex-biased isoforms and sex-biased AS events, we identified over 150 overlapped genes, suggesting that sex-biased isoforms contribute to shaping sex-biased AS patterns. Furthermore, functional enrichment of sex-biased isoforms associated genes revealed distinct differences between sexes. In female embryos, the most enriched functions were negative regulation of transcriptional activities and RNA biosynthetic process, while in male embryos, sex-biased isoform-associated genes were primarily involved in peptide metabolism, translation, and ATP metabolism. Overall, these findings provided novel insights into how sex-biased AS events, isoform expression, and gene-level expression collectively drive sex-specific early development.

## Limitations

The first limitation of this study is sample collection. Due to constraints in sample availability, we only focused on expanded blastocysts for RNA-seq analysis. Future investigations that include embryos from multiple developmental stages would provide a more comprehensive view of sex-specific transcriptomic changes across early development. Another limitation of this study is the use of pooled embryos in each biological replicate. Due to the limited availability of expanded blastocysts, especially female embryos, each RNA-seq sample was composed of two half-cut embryos of the same sex. This pooling strategy ensured sufficient RNA input and reduced the impact of individual outliers, providing a more consistent representation of group-level sex differences. However, pooling may mask inter-individual variability and affect variance estimates in DESeq2. Despite this limitation, the number of pooled replicates (5 male and 6 female) used supports reliable differential expression analysis. Future studies using single-embryo RNA-seq will be important for capturing biological variation at the individual level.

Additionally, the primary aim of this study is to identify transcriptomic differences between female and male blastocysts, including gene expression, alternative splicing, and isoform diversity, and we did not examine epigenetic regulation. Future studies in sex-specific epigenetic modification, specifically DNA methylation, histone modification, and microRNAs, will gain a more integrative understanding of early developmental regulation. Moreover, we used the BO-IVF culture medium, which is widely used for bovine IVF. While our findings suggest that sex-specific differences in developmental speed may be linked to metabolic pathways, we cannot definitively attribute this to specific components such as glucose, as the medium was not designed to isolate the effects of individual nutrients. We recognize that nutrient composition is likely a contributing factor to sex-biased developmental dynamics under in vitro conditions. Therefore, future studies using defined culture media with controlled nutrient levels will be essential to dissect how specific components influence sex-specific developmental trajectories. Furthermore, functional validation was beyond the scope of this study. Although we identified differentially expressed genes and transcription factors, such as PRDM14 and FMR1, in pathways related to the pluripotency regulation and embryo development, it remains unclear whether manipulating these genes or regulators would directly change sex-specific differences during early embryogenesis. Future functional interference studies, including gene knockdown, overexpression, inhibition, or activation experiments on these sex-biased genes or regulators, are necessary to determine the causal roles of these candidate regulators in sex-biased developmental processes.

## Conclusion

The faster development in male embryos compared to females has been observed across multiple species. However, the transcriptomic mechanisms driving these sex-specific differences in early embryo development remain incompletely understood. Through a comprehensive analysis of sex-biased DEGs, AS events, and DEIs in expanded blastocysts, we found that the faster cleaving of male embryos is driven by enhanced metabolic activity, particularly in amino acids and carbon metabolism. In contrast, female blastocysts exhibit slower cleavage, with an enrichment in female-specific developmental processes and pathways. We also identified several sex-specific transcription factors, RBPs, and splicing factors involved in DEG and AS regulation. Moreover, sex-biased AS events and DEIs play a critical role in fine-tuning sex-specific gene expression profiles, contributing to early developmental differences. Additionally, XCU and incomplete XCI appear to influence transcription activity in female embryos, potentially contributing to their developmental difference. This study provides new insights into how chromosomal composition and different levels of transcriptomic regulation contribute to sex differences in bovine early embryonic development, emphasizing the importance of considering sex as a factor in experimental design. Moreover, this study only focused on the expanded blastocyst stage. Future investigation examining earlier or later developmental stages is needed to provide a more comprehensive understanding of sex differences during early reprogramming and development.

## Supplementary Information

Supplemental Material 1: Figure S1. PCA of top 500 most variable genes in samples

Supplemental Material 2: Figure S2. PPI networks among male- and female-biased DEGs and non-DEGs. Genes were ranked along the axis based on the degree of interactions

Supplemental Material 3: Figure S3. Motif calling for female- and male-biased DEGs. A) Motifs at promoter regions of female-biased DEGs. B) Motifs at promoter regions of male-biased DEGs. The motifs are clustered based on sequence similarity

Supplemental Material 4: Figure S4. X:A ratio in bovine blastocysts. The X:A ratio was recalculated in comparison of included or excluded the paralog genes in gene subgroups

Supplemental Material 5: Figure S5. Distribution of Female-biased DEGs on X-chromosome. The left panel was female-biased DEGs, the middle panel was all annotated genes on X-chromosome, and the right panel was the known XCI escapee candidates. Gene density was calculated with 1Mb window. PAR: pseudoautosomal region

Supplemental Material 6: Figure S6. Binding potential of Splicing factors FMR1 and HNRNPH2 on sex-biased skipped exonevents. Motif map showing the potential enrichment of FMR1 and HNRNPH2 binding motifs near the sex-biased alternatively spliced exons. Splicing events were identified using rMATS from a total of 11 RNA-seq samples. The green block indicated the alternative exons in the sex-biased SE events. Negative numbers on the X-axis mark the upstream region of the alternative exons, and the positive numbers mark the downstream region of the alternative exons. The Y-axis shows the binding potential of each splicing factor). The red line indicated the binding potential for female-biased SE events, and the blue line indicated the binding potential for male-biased SE events. Higher peaks indicate greater predicted binding potential of splicing factors at the target regions flanking the alternative exon

Supplemental Material 7: Figure S7. Features of long-read isoforms. A) Illustrations of different categories for long-read isoforms, including full splice match FSM), incomplete splice match, novel in catalog, novel not in catalog, genic Intron, and genic genomic. B) Number of long-read isoforms in each category. C) Number of isoforms per gene based on long-read data. D) The percent of transcripts with annotation supported isoforms, canonical junctions, and splicing junctions with short-read coveragefor the top 4 isoforms categories

Supplemental Material 8: Figure S8. Expression pattern at the gene level and the associated isoforms. Expression profiles are shown for three representative genes:APOE,KDM6A, andGOLM2. For each gene, boxplots display overall gene expression and expression of individual isoforms. Expression quantification was performed using Salmon based on 11 RNA-seq samples. *: padj < 0.05; ns: non-significant. These examples highlight cases where gene-level expression may not reflect isoform-level differences, emphasizing the value of isoform-resolved analysis

Supplemental Material 9: Figure S9. The sex-biased exon usage in *DENND1B* and *DIS3L2*. A) Significant female-biased exon usagein *DENND1B *on negative strand. B) Significant female-biased exon usagein *DIS3L2 *on positive strand. The top line plot showed the exon usage information between sexes, and the purple block below indicated the exons with significantly differential usages between sexes.

Supplemental Material 10: Figure S10. PCR validation of SE events. The SE events were validated in *CEP350*, *ZMYND11*, and *WWC2*. The left three panels were the gel image of the PCR products, while the right panels were the example to show the skipped exon highlighted in green. F1, F2, and F3 are female embryos; while M1, M2, and M3 are male embryos.

Supplemental Material 11: Figure S11. AS validation. This bar plot compared the inclusion level between the PCR validation and RNA-seq data. AS events between sexes were compared via the inclusion levels, which is the ratio of the expression level of the transcripts with the skipped exon to the expression level of the total he transcripts. The expression level of PCR validation was calculated based on the brightness of the band with Image-J

Supplemental Material 12: Table S1. Primer information for embryo determination. Table S2. Primer information for sex-biased DEG validation. Table S3. Primer information for alternative splicing validation

Supplemental Material 13: Table S4. Sex ratio of bovine IVF embryos and summary statistics of RNA-seq data

Supplemental Material 14: Table S5. Differentially expressed genes and functional enrichment

Supplemental Material 15: Table S6. Overlapped genes between sex-biased DEGs and AS genes and functional enrichment

Supplemental Material 16: Table S7. Sex-biased alternative splicing events

Supplemental Material 17: Table S8. Protein domains affected by sex-biased SE events and functional enrichment

Supplemental Material 18: Table S9. Sex-biased isoforms and functional enrichment

Supplemental Material 19: Table S10. Overlapping genes among sex-biased DEGs, differential alternative splicing genes, and differential isoform-related genes

Supplemental Material 20: Table S11. Differential exon usage between sexes

## Data Availability

The RNA-seq raw data have been deposited at NCBI Gene Expression Omnibus database GSE290265.The current study did not generate new code; all codes used for analysis in this study can be found according to corresponding references.
