# Supplementary figures and images for: Sex-biased transcriptome in in vitro produced bovine early embryos
Source: Cell Biosci. 2025 Aug 27;15:123. doi: 10.1186/s13578-025-01459-x (PMC12392601; doi:10.1186/s13578-025-01459-x)

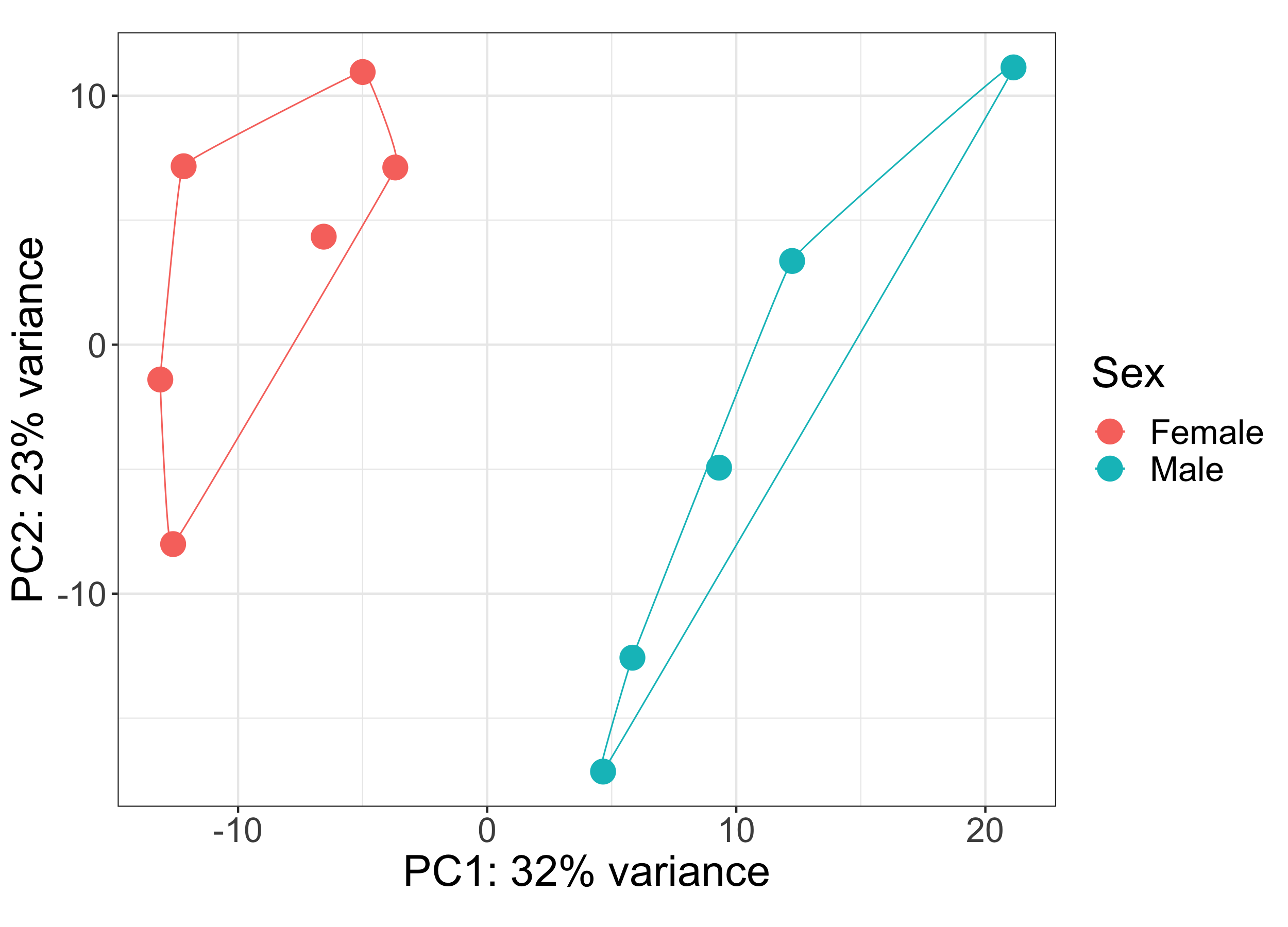

Supplement: Supplementary file 1 — Supplemental Material 1: Figure S1. PCA of top 500 most variable genes in samples [file 13578_2025_1459_MOESM1_ESM.tif]

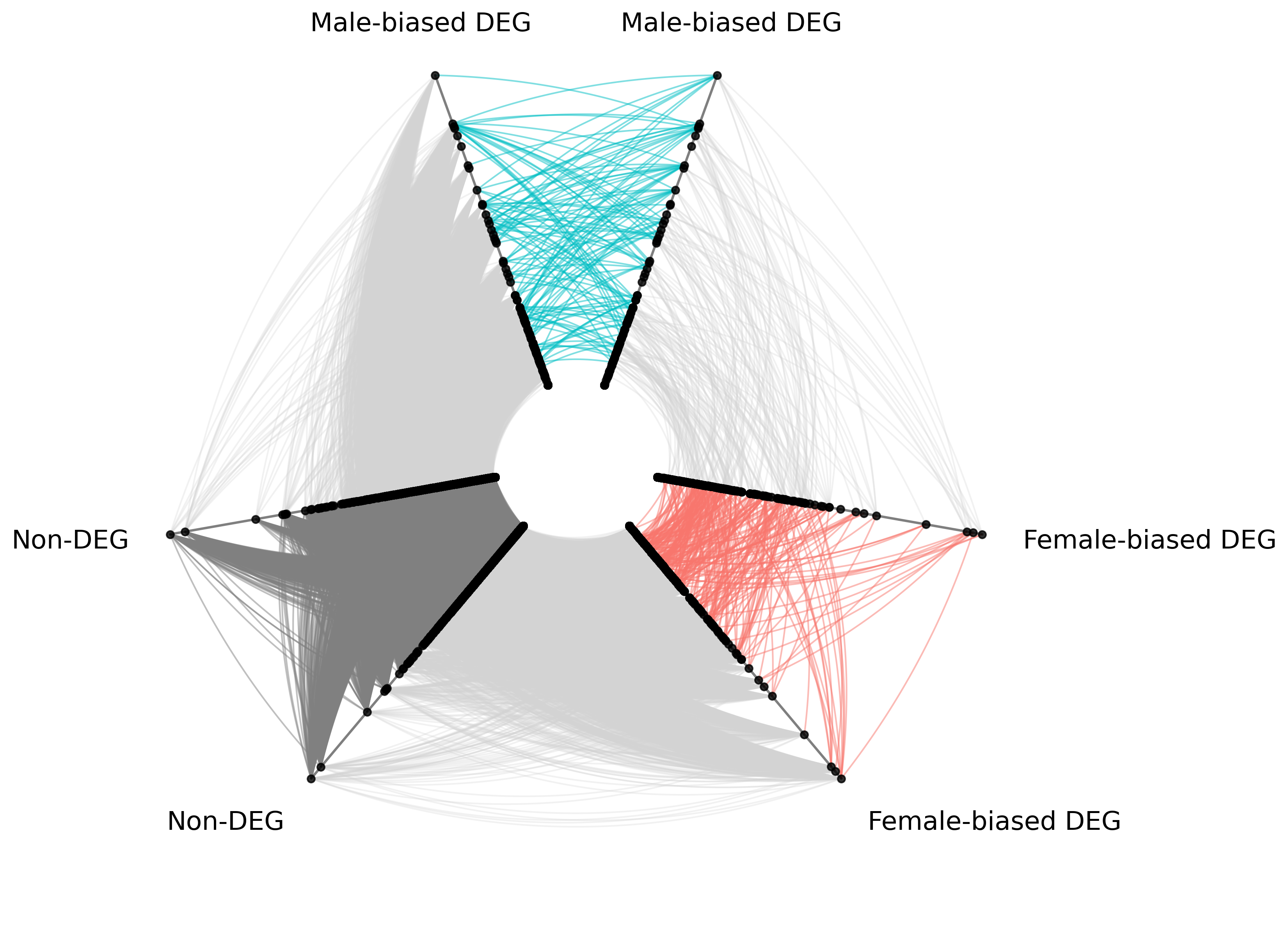

Supplement: Supplementary file 2 — Supplemental Material 2: Figure S2. PPI networks among male- and female-biased DEGs and non-DEGs. Genes were ranked along the axis based on the degree of interactions [file 13578_2025_1459_MOESM2_ESM.tif]

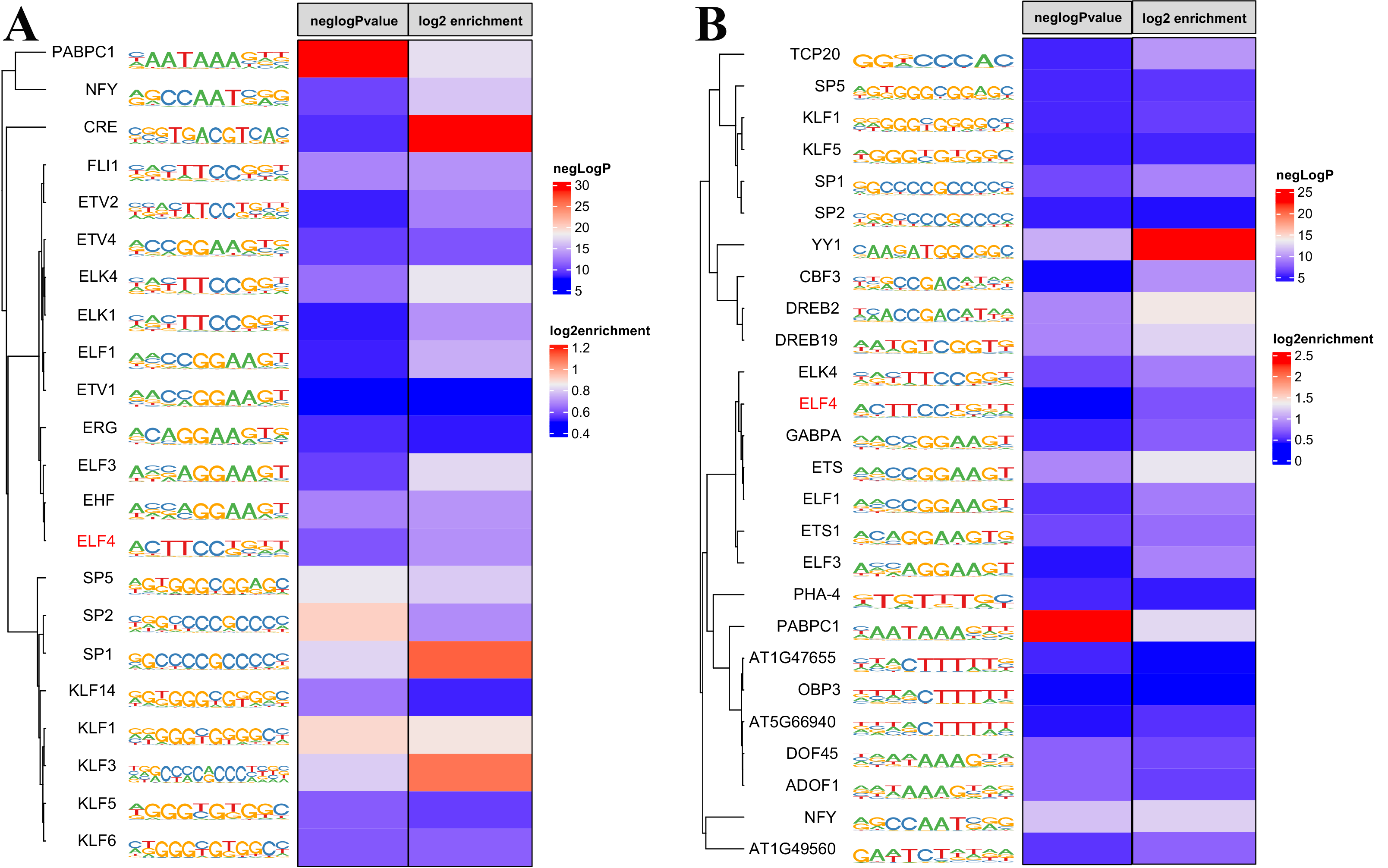

Supplement: Supplementary file 3 — Supplemental Material 3: Figure S3. Motif calling for female- and male-biased DEGs. A) Motifs at promoter regions of female-biased DEGs. B) Motifs at promoter regions of male-biased DEGs. The motifs are clustered based on sequence similarity [file 13578_2025_1459_MOESM3_ESM.tif]

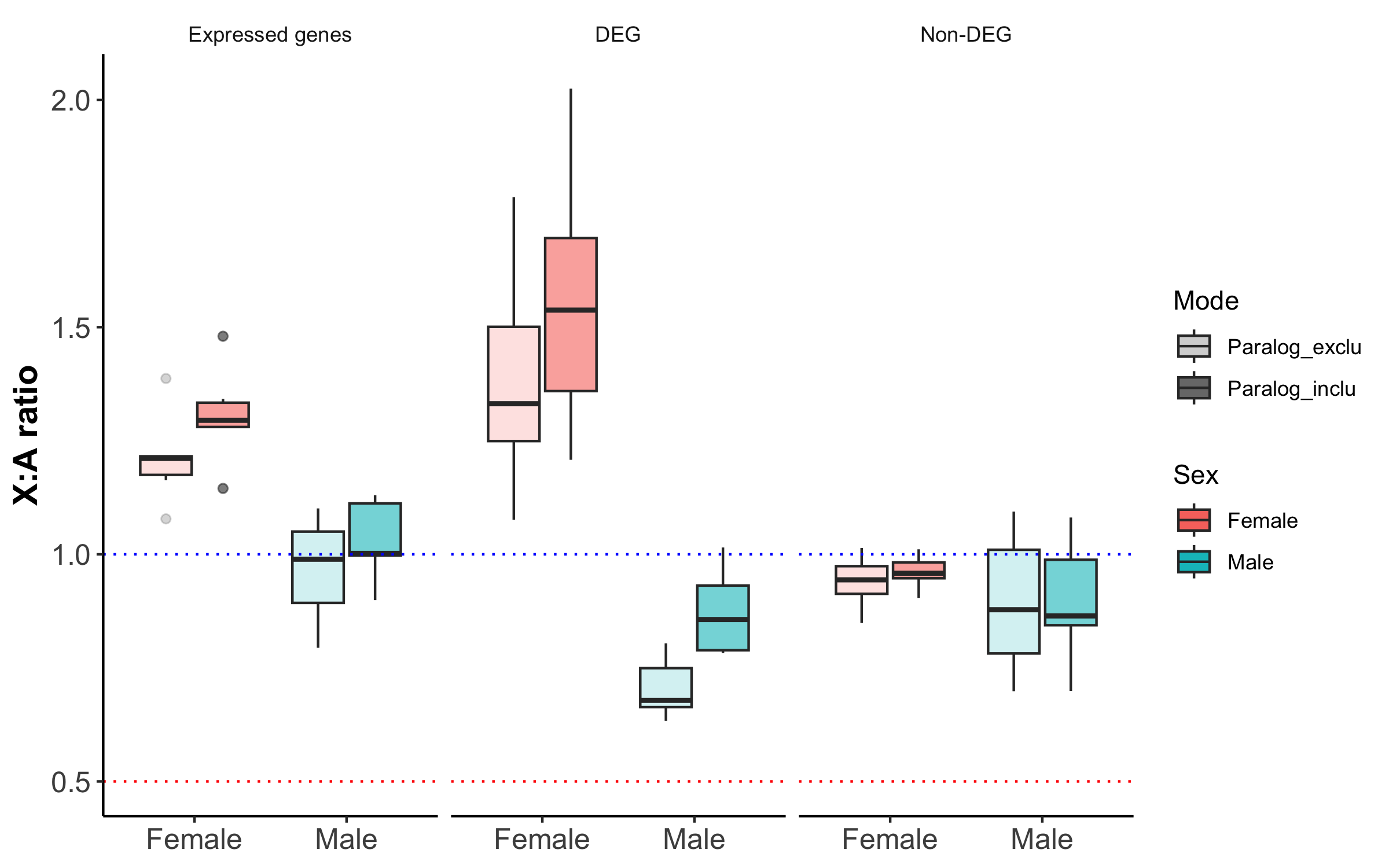

Supplement: Supplementary file 4 — Supplemental Material 4: Figure S4. X:A ratio in bovine blastocysts. The X:A ratio was recalculated in comparison of included or excluded the paralog genes in gene subgroups [file 13578_2025_1459_MOESM4_ESM.tif]

DEGs

All genes

Escapee genes

Density

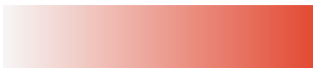

Low

High

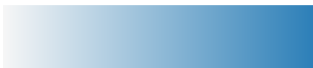

Low

High

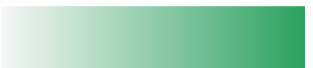

Low

High

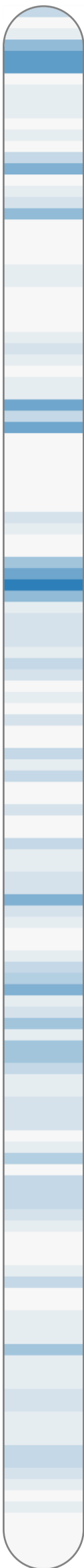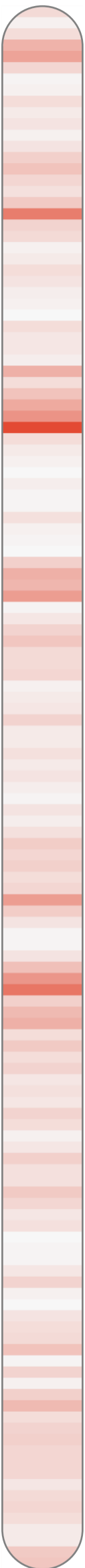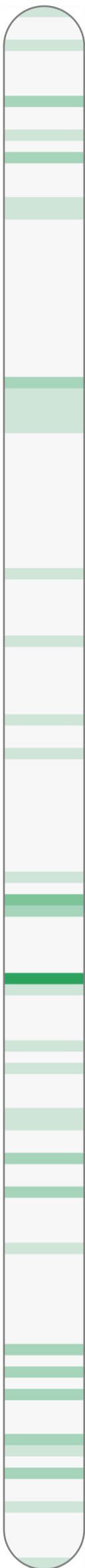

XIST

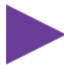

PAR

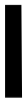

Chromosome X

Supplement: Supplementary file 5 — Supplemental Material 5: Figure S5. Distribution of Female-biased DEGs on X-chromosome. The left panel was female-biased DEGs, the middle panel was all annotated genes on X-chromosome, and the right panel was the known XCI escapee candidates. Gene density was calculated with 1Mb window. PAR: pseudoautosomal region [file 13578_2025_1459_MOESM5_ESM.pdf]

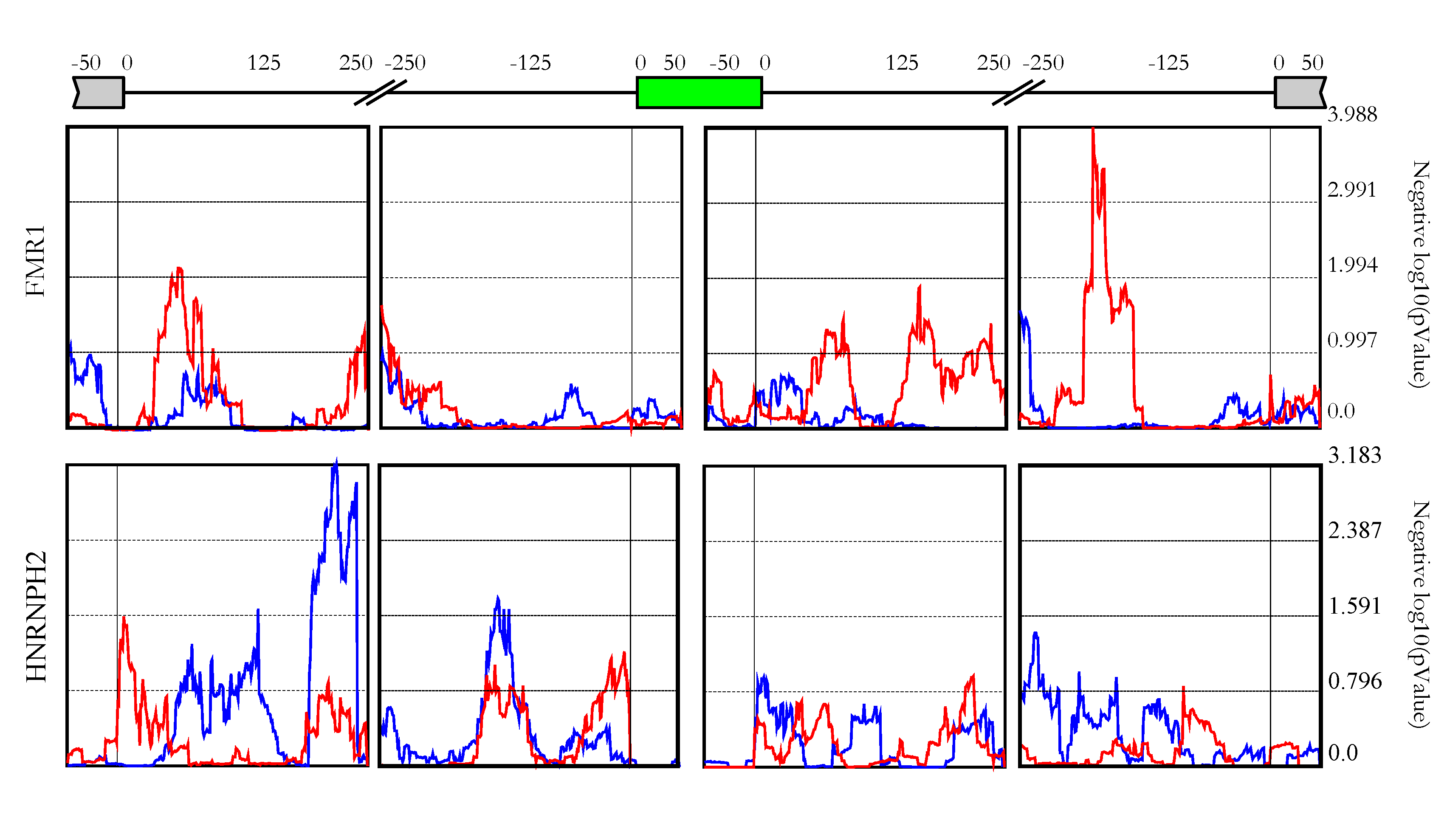

Supplement: Supplementary file 6 — Supplemental Material 6: Figure S6. Binding potential of Splicing factors FMR1 and HNRNPH2 on sex-biased skipped exonevents. Motif map showing the potential enrichment of FMR1 and HNRNPH2 binding motifs near the sex-biased alternatively spliced exons. Splicing events were identified using rMATS from a total of 11 RNA-seq samples. The green block indicated the alternative exons in the sex-biased SE events. Negative numbers on the X-axis mark the upstream region of the alternative exons, and the positive numbers mark the downstream region of the alternative exons. The Y-axis shows the binding potential of each splicing factor). The red line indicated the binding potential for female-biased SE events, and the blue line indicated the binding potential for male-biased SE events. Higher peaks indicate greater predicted binding potential of splicing factors at the target regions flanking the alternative exon [file 13578_2025_1459_MOESM6_ESM.tif]

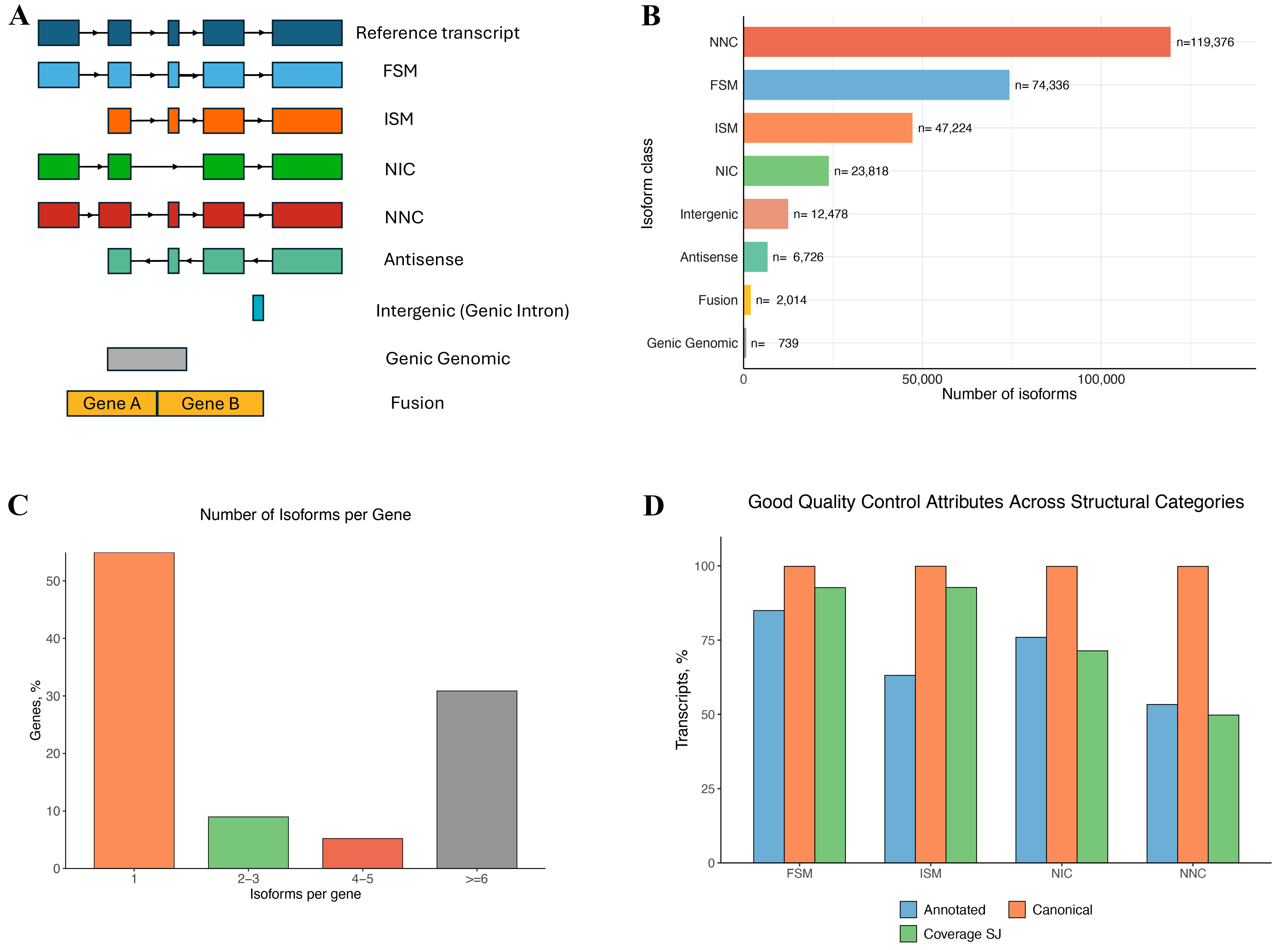

Supplement: Supplementary file 7 — Supplemental Material 7: Figure S7. Features of long-read isoforms. A) Illustrations of different categories for long-read isoforms, including full splice match FSM), incomplete splice match, novel in catalog, novel not in catalog, genic Intron, and genic genomic. B) Number of long-read isoforms in each category. C) Number of isoforms per gene based on long-read data. D) The percent of transcripts with annotation supported isoforms, canonical junctions, and splicing junctions with short-read coveragefor the top 4 isoforms categories [file 13578_2025_1459_MOESM7_ESM.tif]

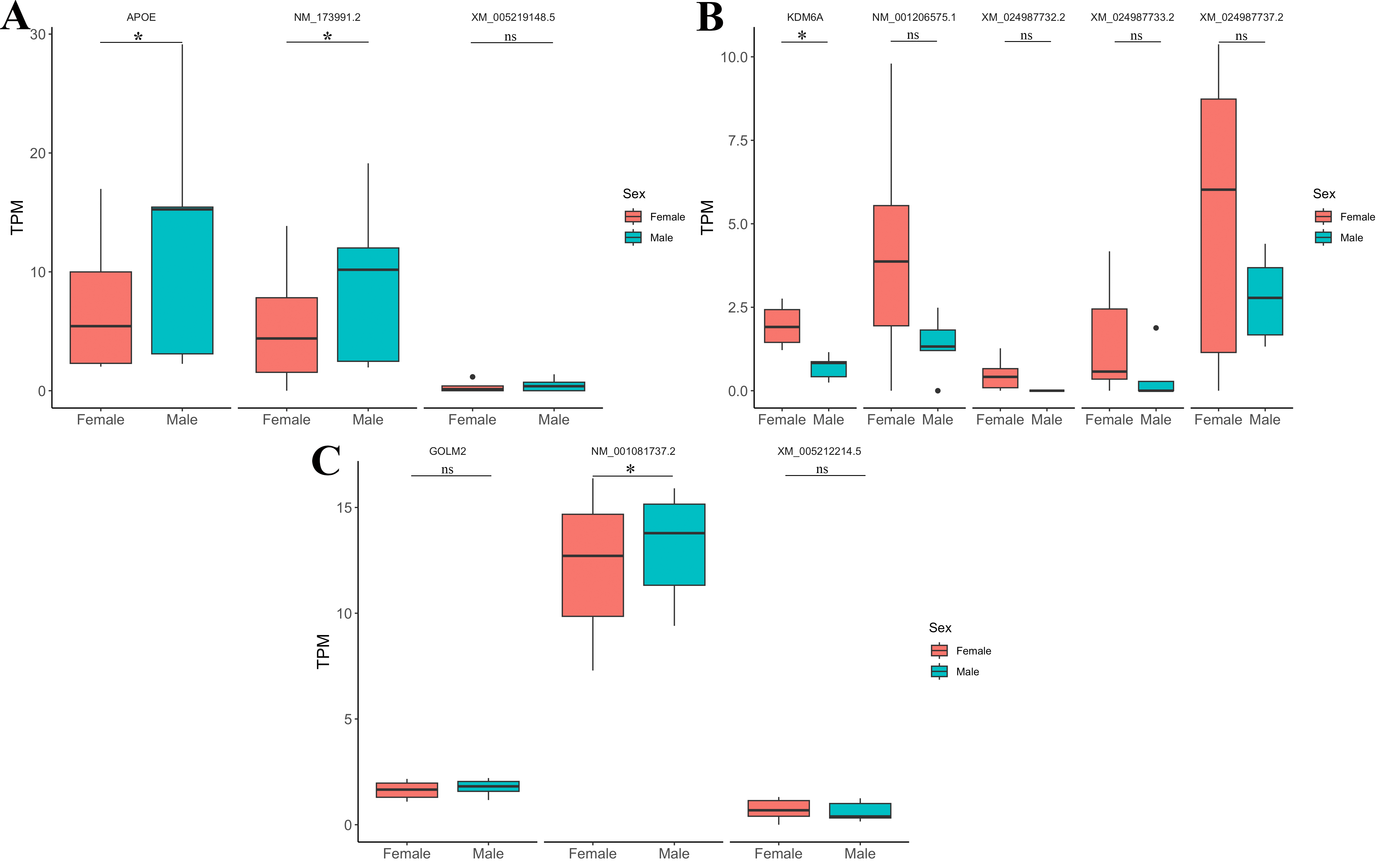

Supplement: Supplementary file 8 — Supplemental Material 8: Figure S8. Expression pattern at the gene level and the associated isoforms. Expression profiles are shown for three representative genes:APOE,KDM6A, andGOLM2. For each gene, boxplots display overall gene expression and expression of individual isoforms. Expression quantification was performed using Salmon based on 11 RNA-seq samples. *: padj < 0.05; ns: non-significant. These examples highlight cases where gene-level expression may not reflect isoform-level differences, emphasizing the value of isoform-resolved analysis [file 13578_2025_1459_MOESM8_ESM.tif]

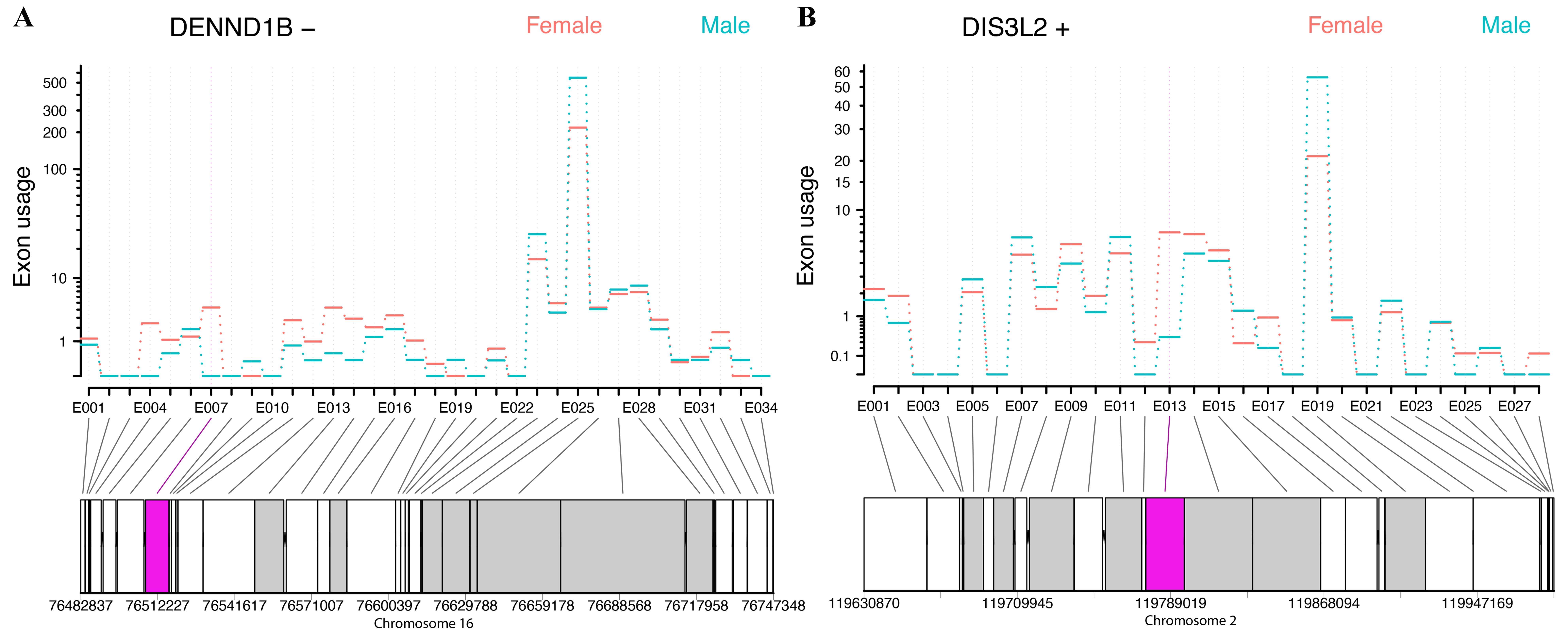

Supplement: Supplementary file 9 — Supplemental Material 9: Figure S9. The sex-biased exon usage in DENND1B and DIS3L2. A) Significant female-biased exon usagein DENND1B on negative strand. B) Significant female-biased exon usagein DIS3L2 on positive strand. The top line plot showed the exon usage information between sexes, and the purple block below indicated the exons with significantly differential usages between sexes. [file 13578_2025_1459_MOESM9_ESM.tif]

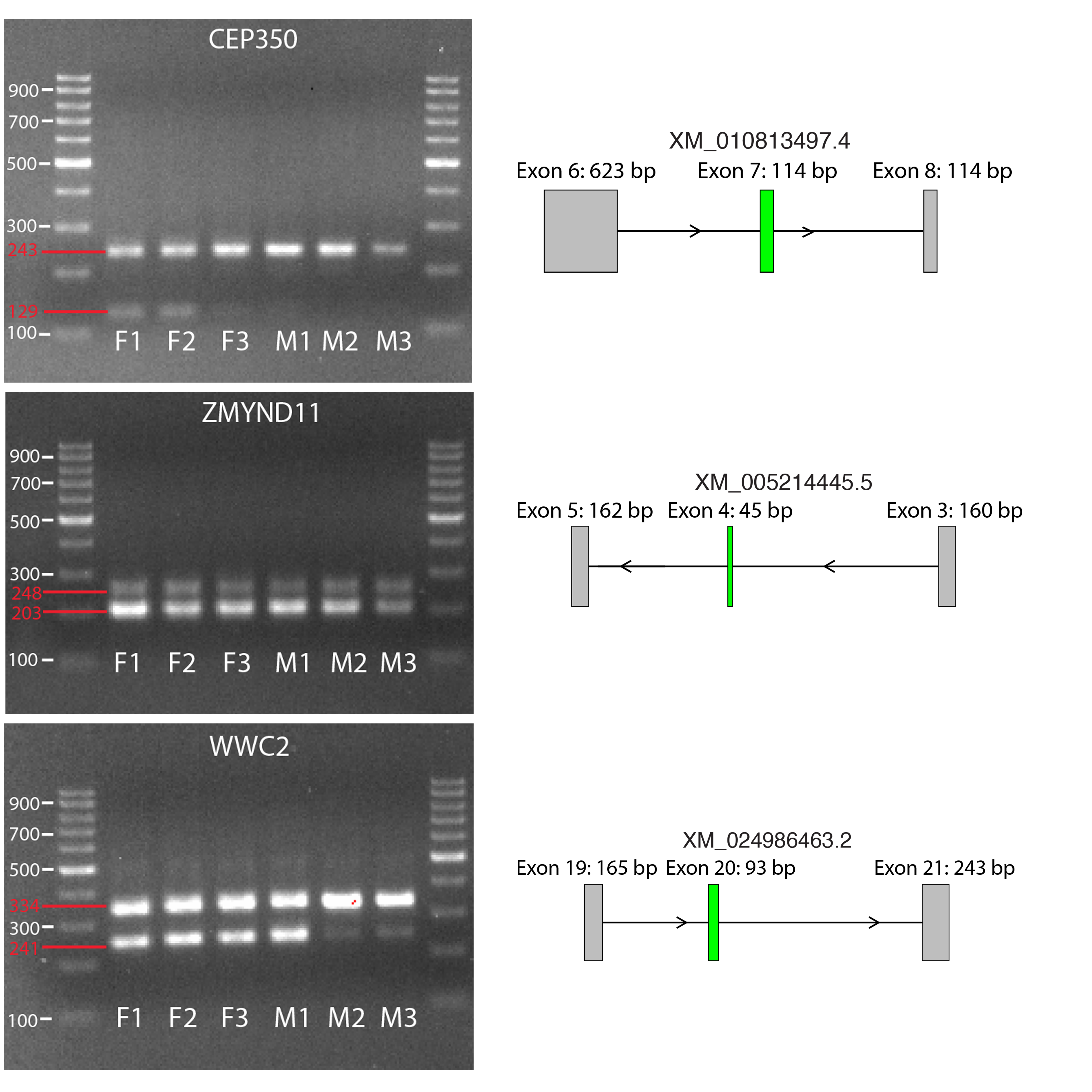

Supplement: Supplementary file 10 — Supplemental Material 10: Figure S10. PCR validation of SE events. The SE events were validated in CEP350, ZMYND11, and WWC2. The left three panels were the gel image of the PCR products, while the right panels were the example to show the skipped exon highlighted in green. F1, F2, and F3 are female embryos; while M1, M2, and M3 are male embryos. [file 13578_2025_1459_MOESM10_ESM.tif]

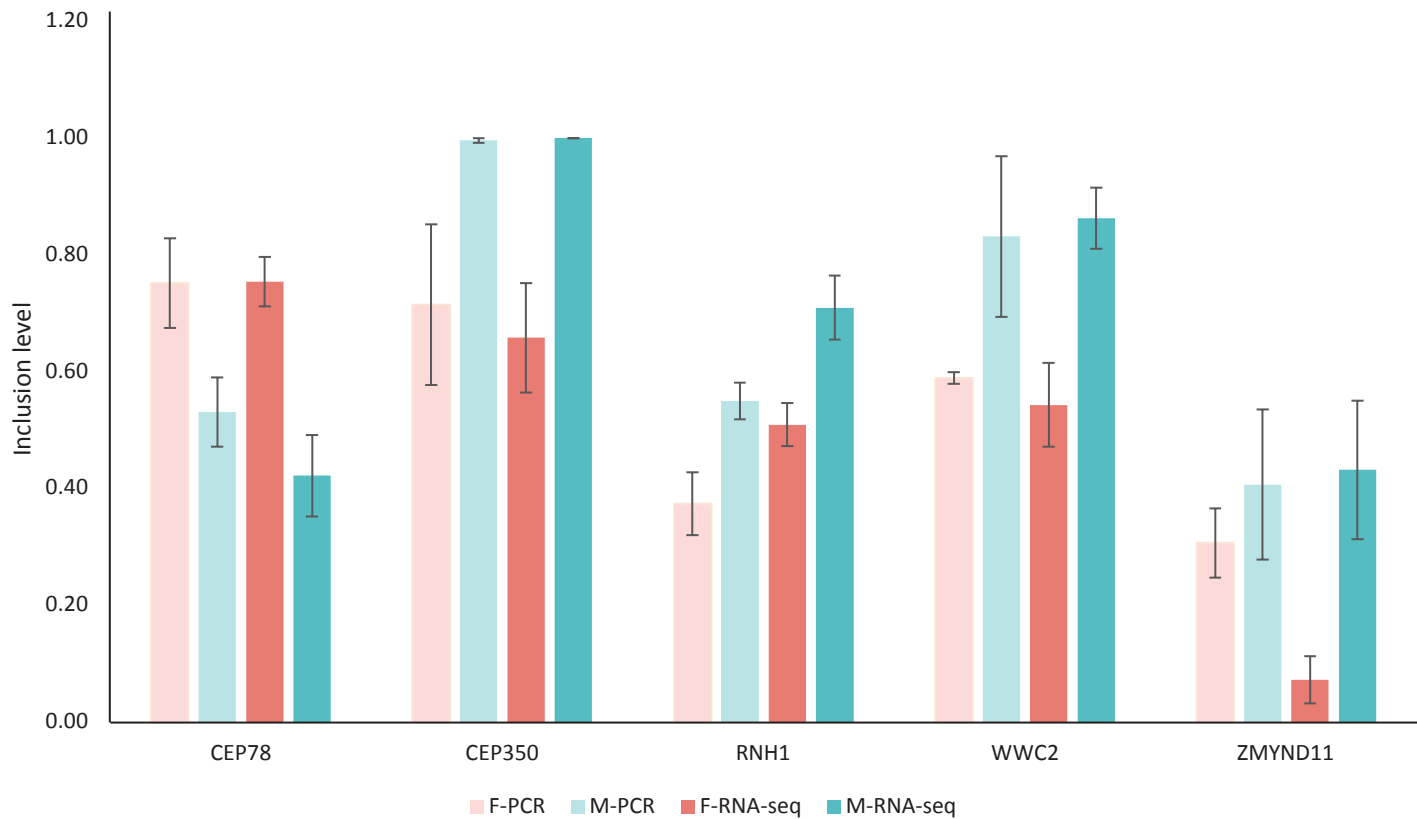

Supplement: Supplementary file 11 — Supplemental Material 11: Figure S11. AS validation. This bar plot compared the inclusion level between the PCR validation and RNA-seq data. AS events between sexes were compared via the inclusion levels, which is the ratio of the expression level of the transcripts with the skipped exon to the expression level of the total he transcripts. The expression level of PCR validation was calculated based on the brightness of the band with Image-J [file 13578_2025_1459_MOESM11_ESM.pdf]
